# Digoxin for reduction of circulating tumor cell cluster size in metastatic breast cancer: a proof-of-concept trial

**DOI:** 10.1038/s41591-024-03486-6

**Published:** 2025-01-24

**Authors:** Christian Kurzeder, Bich Doan Nguyen-Sträuli, Ilona Krol, Alexander Ring, Francesc Castro-Giner, Manuel Nüesch, Simran Asawa, Yu Wei Zhang, Selina Budinjas, Ana Gvozdenovic, Maren Vogel, Angela Kohler, Cvetka Grašič Kuhar, Fabienne D. Schwab, Viola Heinzelmann-Schwarz, Walter Paul Weber, Christoph Rochlitz, Denise Vorburger, Heike Frauchiger-Heuer, Isabell Witzel, Andreas Wicki, Gabriela M. Kuster, Marcus Vetter, Nicola Aceto

**Affiliations:** 1 Department of Gynecology and Gynecologic Oncology, University Hospital Basel, University of Basel, Basel, Switzerland; 2 Department of Gynecology, University Hospital Zurich, University of Zurich, Zurich, Switzerland; 3 Department of Biology, Institute of Molecular Health Sciences, Swiss Federal Institute of Technology, Zurich, Switzerland; 4 Department of Medical Oncology and Hematology, University Hospital Zurich, University of Zurich, Zurich, Switzerland; 5 Center of Oncology and Hematology, Medical University Clinic, Kantonsspital Baselland, Liestal, Switzerland; 6 Breast Center, University Hospital Basel, University of Basel, Basel, Switzerland; 7 Department of Medical Oncology, Institute of Oncology Ljubljana and Faculty of Medicine, University of Ljubljana, Ljubljana, Slovenia; 8 Department of Medical Oncology, University Hospital Basel, Basel, Switzerland; 9 Department of Cardiology, University Hospital Basel, University of Basel, Basel, Switzerland; 10 Cancer Center Baselland, Medical University Clinic, Kantonsspital Baselland, Liestal, Switzerland

**Keywords:** Breast cancer, Metastasis

## Abstract

The presence of circulating tumor cell (CTC) clusters is associated with disease progression and reduced survival in a variety of cancer types. In breast cancer, preclinical studies showed that inhibitors of the Na^+^/K^+^ ATPase suppress CTC clusters and block metastasis. Here we conducted a prospective, open-label, proof-of-concept study in women with metastatic breast cancer, where the primary objective was to determine whether treatment with the Na^+^/K^+^ ATPase inhibitor digoxin could reduce mean CTC cluster size. An analysis of nine patients treated daily with a maintenance digoxin dose (0.7–1.4 ng ml^−1^ serum level) revealed a mean cluster size reduction of −2.2 cells per cluster upon treatment (*P* = 0.003), meeting the primary endpoint of the study. Mechanistically, transcriptome profiling of CTCs highlighted downregulation of cell–cell adhesion and cell-cycle-related genes upon treatment with digoxin, in line with its cluster-dissolution activity. No treatment-related adverse events occurred. Thus, our data provide a first-in-human proof of principle that digoxin treatment leads to a partial CTC cluster dissolution, encouraging larger follow-up studies with refined Na^+^/K^+^ ATPase inhibitors and that include clinical outcome endpoints. ClinicalTrials.gov identifier: NCT03928210.

## Abstract (web summary)

This proof-of-concept phase 1 trial shows that the size of circulating tumor cell clusters (which can promote metastatic spread) can be reduced by treatment with the Na^+^/K^+^ ATPase inhibitor digoxin in patients with metastatic breast cancer.

## Main

Breast cancer is the most diagnosed cancer among women globally^1^. In the past decade, multimodal approaches and innovative therapies have transformed the outlook of this lethal disease, leading to gains in patient survival^2^. Despite these advances, nearly 685,000 women die of breast cancer each year worldwide^1^, largely due to the development of incurable distant metastases to vital organs^3^. In this context, a potentially critical factor may lie within the underlying principles of most anticancer drugs. Standard-of-care treatments are typically developed on the basis of their cytotoxic activity and are not necessarily designed to interfere with metastasis-relevant mechanisms^4,5^. Consequently, there is an intriguing yet uncharted opportunity for the development of metastasis-targeted agents that disrupt the causes of metastasis themselves^4,5^.

Circulating tumor cells (CTCs) are living cells that are shed from both primary and metastatic lesions into the bloodstream, acting as metastatic pioneers^6^. The presence of CTCs has been firmly established to be predictive of poor prognosis in patients with breast cancer^7^. Recent studies by us and others demonstrated that clusters of CTCs, defined as multicellular aggregates of cancer cells alone (homotypic) or in liaison with immune cells (heterotypic), have a substantially higher metastatic capacity and a stronger association with a poor prognosis than single CTCs^8–10^. Preclinical studies further revealed unique biological properties and vulnerabilities of these clusters, such as stem-like and proliferation features dependent upon cell–cell adhesion integrity^8,11^. A screen with 2,486 US Food and Drug Administration-approved drugs demonstrated that Na^+^/K^+^ ATPase inhibitors, such as cardiac glycosides, effectively dissolve CTC clusters into single cells, leading to metastasis suppression in mouse models of breast cancer^11^. Consequently, the Digoxin Induced Dissolution of CTC Clusters (DICCT) trial has been set up as a multicentric, prospective, first-in-human proof-of-concept, single-arm, therapeutic exploratory phase 1 study aimed to examine whether the Na^+^/K^+^ ATPase inhibitor digoxin could disrupt CTC clusters in patients with metastatic breast cancer at dose levels that are safe and well tolerated (NCT03928210; DICCT/Swiss-GO-07).

**Fig. 1:**
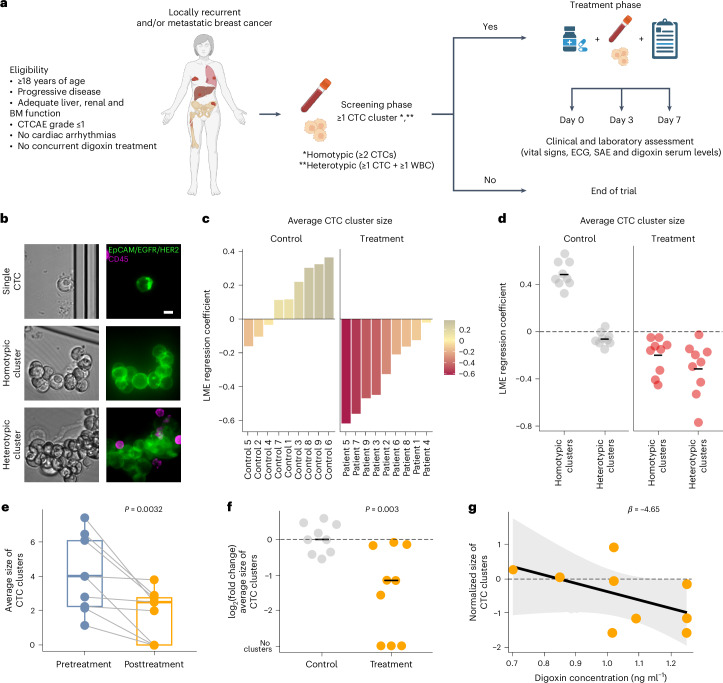
Study design and digoxin treatment response assessment. **a**, Study flow chart. **b**, Representative images of a single CTC and homotypic and heterotypic CTC clusters (scale bar, 10 µm), stained with EpCAM, HER2 and EGFR (green) and CD45 (magenta). **c**,**d**, LME random coefficients showing a negative association between treatment and the average size of all CTC clusters (**c**) and among homotypic (regression coefficient −0.20, 95% CI −0.76 to 0.35) or heterotypic (regression coefficient −0.31, 95% CI −1.21 to 0.59) clusters, separately (**d**). LME coefficients are also shown for control nonrandomized patients, not receiving digoxin therapy (regression coefficient 0.48, 95% CI −0.10 to 1.07 for homotypic clusters; regression coefficient −0.06, 95% CI −0.70 to 0.58 for heterotypic clusters). The cross bar in **d** represents the LME fixed-effect coefficient. **e**, The average cluster size at baseline and posttreatment (day 3 or day 7) paired by patient (*n* = 9). The boxes represent the lower quantile, median and upper quantile. The vertical lines show the range of values, and the gray lines connect paired values. *P* values were calculated using the one-sided paired *t*-test. **f**, The fold change of the average CTC cluster size post- over pretreatment in treated patients. In control patients, the fold change of the average CTC cluster size at day 3 or day 7 (according to smaller cluster size) over baseline is shown. Each point represents an individual patient, and the cross bar represents the median. *P* values were calculated using the one-sided Wilcoxon rank-sum test. **g**, Negative association between digoxin levels and normalized size of CTC clusters at day 7 (linear regression *P* = 0.14, *β* = −4.65). The points represent individual patients, the line represents the linear regression model and the shaded area represents the 95% CI of the fitted line. CTCAE, Common Terminology Criteria for Adverse Events; BM, bone marrow; ECG, electrocardiogram; SAE, serious adverse event. Panel **a** was created with BioRender.com. Source data

**Extended Data Fig. 1:**
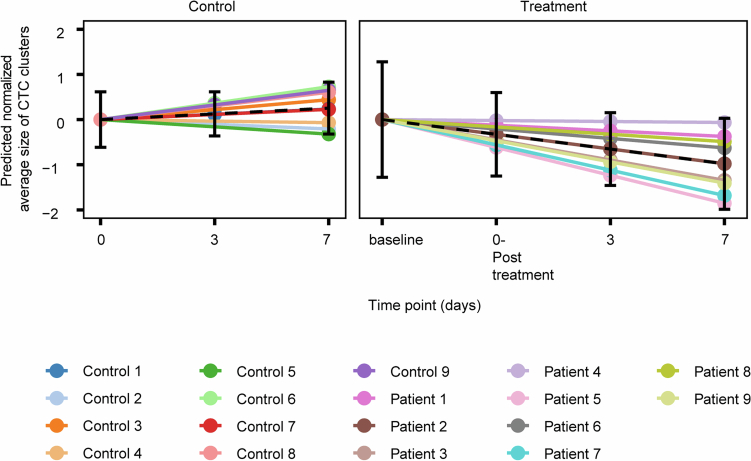
Predicted average size of circulating tumor cell (CTC) clusters for each control and treated patient using linear mixed effect. The lines represent the regression line for the linear model for each patient and the points represent the predicted values at each timepoint according to the model. Error bars indicate the 95% confidence interval.

**Extended Data Fig. 2:**
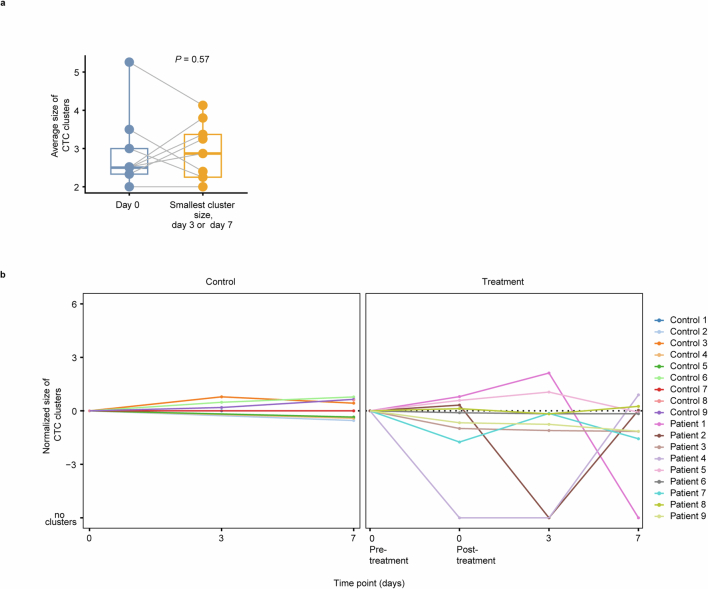
CTC cluster size in control and digoxin-treated patients. (**a**) Average cluster size at baseline and at follow up (smallest cluster size, day 3 or day 7) paired by patient (control cohort, *n* = 9). Boxes represent lower quantile, median and upper quantile. Vertical lines indicate the range of values, grey lines connect paired values. (**b**) Average CTC cluster size across timepoints normalized by the average CTC cluster size at baseline (0) in control and treated patients. Values in Y-axis are represented in log2 scale. The colored lines indicate individual patients.

**Extended Data Fig. 3:**
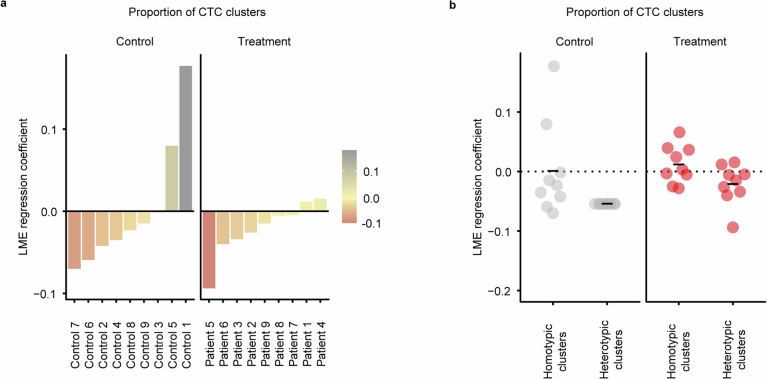
CTC proportions in control and digoxin-treated patients. Linear mixed effects (LME) random coefficients showing the association between assessment time points and the proportion of all types of circulating tumor cells (CTC) clusters (secondary endpoint) per control and treated patients (**a**) or split into heterotypic and homotypic clusters (**b**). The cross bar in **b** represents the LME fixed effect coefficient.

The primary objective of the study was to assess the effect of digoxin on CTC cluster size in patients with metastatic breast cancer. Of note, the size of CTC clusters, rather than their general abundance, best reflects cluster-dissolution properties. Secondary objectives included the effect of digoxin on the overall abundance of CTC clusters, the kinetics of CTC cluster dissolution and the dose–response relationship of the effect. Patients aged 18 years or older with locoregionally recurrent or metastatic breast cancer with progressive disease not amenable to treatments with curative intent were eligible for study inclusion. A total of 58 patients were screened by means of peripheral blood sampling and CTC cluster assessment. Of these, 11 patients resulted positive for CTC clusters at baseline, were enrolled in DICCT and received digoxin at 0.125–0.250 mg per day (intention-to-treat population) (Fig. 1a). Among these, two patients were excluded from the study: one because of the inability to reach the target serum level and another because of a digoxin-unrelated adverse event. Nine patients (*n* = 9) received daily digoxin doses for 7 days and reached target serum levels ≥0.7 ng ml^−1^ in the per-protocol population. Separately, nine patients (*n* = 9) with CTC clusters and matched clinical characteristics were nonrandomly assigned to the untreated control group, where CTC cluster size and composition were assessed over a 1-week period with the purpose to examine pathophysiological CTC cluster size fluctuations in patients not treated with digoxin. All patients (comprising both treated and control cohorts) were enrolled between July 2020 and July 2024, and their baseline clinical characteristics are presented in Table 1 and Extended Data Tables 1 and 2. Blood samples were collected in EDTA tubes at day 0 (immediately before digoxin treatment as well as 2 h after treatment), day 3 and day 7 and processed within 4 h with the US Food and Drug Administration-cleared Parsortix device. Upon microfluidic entrapment and immunofluorescence staining, single CTCs and homotypic and heterotypic clusters were identified and enumerated (Fig. 1b). The distribution of size, number and proportion of homotypic and heterotypic CTC clusters varied between time points and individual patients (Extended Data Tables 3 and 4). Mean cluster size at baseline was 2.9 and 2.5 cells for homotypic clusters and 3.5 and 4.7 for heterotypic clusters in treated and untreated control patients, respectively (Extended Data Tables 3 and 4). Despite the expected variability when sampling relatively small volumes of peripheral blood, a linear mixed-effects (LME) model analysis suggested an overall reduction in cluster size over the 7-day digoxin treatment period (regression coefficient −0.33, 95% confidence interval (CI) −0.89 to 0.24) (Fig. 1c and Extended Data Fig. 1), evident in both homotypic and heterotypic clusters (Fig. 1d). LME coefficients were also calculated for control patients not receiving digoxin therapy, and in contrast to the digoxin-treated cohort, no decrease in cluster size was observed (regression coefficient 0.13, 95% CI −0.25 to 0.51) (Fig. 1c,d and Extended Data Fig. 1). The study met its primary endpoint with a significant reduction in average cluster size between pre- and posttreatment (mean difference of −2.2 cells, one-sided paired *t*-test *P* value 0.0032) (Fig. 1e), where posttreatment values were taken either at day 3 or at day 7 according to best response, given well-known challenges in digoxin dosing^12^. Of note, the significant reduction of average cluster CTC size is observed exclusively in treated patients and not in control samples analyzed with the same metric (Fig. 1e,f and Extended Data Fig. 2a,b). Interestingly, despite the small treated cohort, a numerical trend toward a higher digoxin serum concentration and stronger reduction in the average cluster size at day 7 was observed (secondary endpoint) (linear regression coefficient −4.65, *P* = 0.14; Fig. 1g), suggesting that higher digoxin serum levels or more potent Na^+^/K^+^ ATPase inhibitors could be more effective in cluster dissolution. The proportion of single CTCs and CTC clusters was not significantly affected by digoxin treatment at the given concentration (Extended Data Fig. 3). The study treatment was well tolerated, and no adverse events related to digoxin treatment occurred.

**Table 1:** Baseline clinical patient characteristics

	Control (*n* = 9)	Treated (*n* = 9)
**Age at diagnosis (years)**	50.0 (32.0, 68.0)	51.0 (42.0, 83.0)
**Age at enrollment (years)**	55.0 (35.0, 83.0)	59.0 (43.0, 83.0)
**ER (%)**	90.0 (0.0, 100.0)	90.0 (0.0, 100.0)
**PR (%)**	50.0 (0.0, 95.0)	0.0 (0.0, 80.0)
**HER2 amplification**	3 (33.3%)	0 (0.0%)
**Ki67 (%)**	37.5 (10.0, 50.0)	37.5 (7.5, 80.0)
**Histology**		
Ductal	7 (77.8%)	6 (66.7%)
Lobular	1 (11.1%)	3 (33.3%)
Mucinous/neuroendocrine	1 (11.1%)	0 (0.0%)
**Metastasis sites**		
Liver	6 (66.7%)	5 (55.6%)
Lung	5 (55.6%)	3 (33.3%)
Bone	7 (77.8%)	9 (100.0%)
Others	7 (77.8%)	8 (88.9%)
**Number of previous treatment lines**	2.0 (1.0, 5.0)	1.0 (0.0, 9.0)

The table presents the age (years) at primary diagnosis, age (years) at CTC enumeration, subtype of most recent biopsy (percentage of estrogen receptor (ER)-positive cells, percentage of progesterone receptor (PR)-positive cells, percentage of Ki67-positive cells and HER2 amplification status), histologic subtype (ductal, lobular and mucinous/neuroendocrine), site of metastasis and number of previous systemic treatment lines in nonrandomized cohorts of control and digoxin-treated patients. The values in the table represent the median (range) for numeric variables and counts (percentage) for categorical variables.

**Extended Data Fig. 4:**
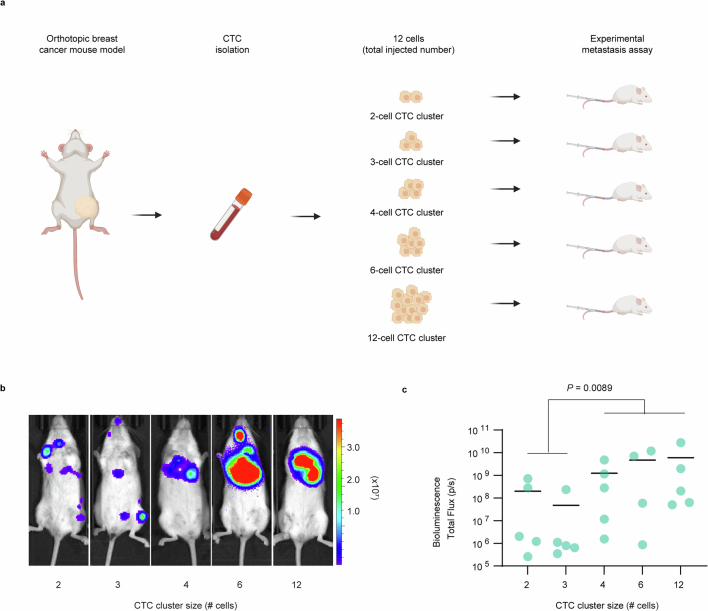
Metastatic potential as a function of different CTC cluster sizes. (**a**) Schematic illustration of the experimental design. (**b**) Representative images of bioluminescent signals in mice within specified experimental groups. (**c**) Plot showing normalized bioluminescent signal across experimental groups (minimum *n* = 4 per group, where points represent individual mice) three weeks after tumor cell injection. *P* = 0.0089 by two-sided Mann-Whitney test. The cross bar represents the mean. Panel (**a**) was created with BioRender (https://biorender.com).

To delineate how CTC cluster size affects disease outcomes, we next conducted animal studies. We injected 4T1 breast cancer cells into the mammary fat pad of NOD.Cg-*Prkdc*^*scid*^ *Il2rg*^*tm1Wjl*^/SzJ (NSG) mice, and upon tumor development, spontaneously generated CTC clusters of different sizes were individually isolated. A total of 12 cells made by clusters of different sizes (2-cell, 3-cell, 4-cell, 6-cell and 12-cell clusters) were intravenously injected into tumor-free recipient mice to measure their direct metastatic ability as a function of their size. Interestingly, CTC clusters of at least four cells exhibited higher metastatic potential compared with smaller clusters, as determined by bioluminescence imaging (two-sided Mann–Whitney test *P* value 0.0089) (Extended Data Fig. 4).

**Extended Data Fig. 5:**
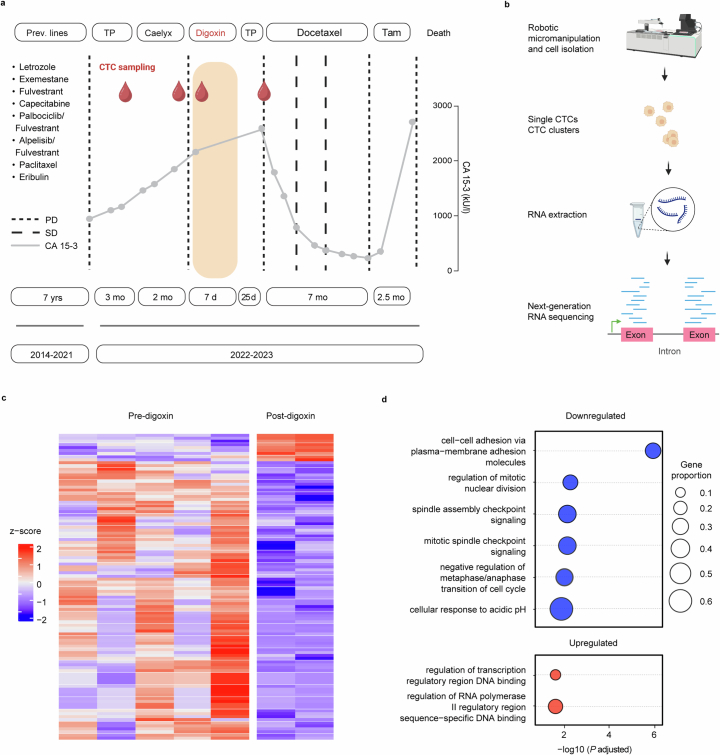
Longitudinal RNA sequencing of CTCs during digoxin treatment. (**a**) The diagram shows the clinical timeline of patient nr. 5, including treatment history (*boxes*), radiological assessment (*dashed lines*) and serum tumor marker (CA 15-3) (*grey*). (**b**) Workflow for molecular analysis, including microfluidic separation and capture, robotic micromanipulation, pooling of circulating tumor cells (CTCs) into individual tubes and RNA sequencing. (**c**) Heatmap displaying differentially expressed genes (DESeq Wald test adjusted *P* value < 0.05) between CTC pools obtained prior to digoxin intake (*n* = 5 pre-digoxin pools) and post-digoxin intake (*n* = 2 post-digoxin pools). Values are displayed as gene-scaled (z-score) log2 counts per million mapped reads after normalization. (**d**) Enriched pathways among downregulated (*top*) and upregulated *(bottom*) genes of CTCs post-digoxin intake (over-representation analysis adjusted *P* value < 0.05). Shown is a reduced list of ontologies after simplification using semantic similarity. TP, treatment pause; PD, progressive disease; SD, stable disease; TAM, tamoxifen. Panels (**a**) and (**b**) were created with BioRender (https://biorender.com).

**Extended Data Fig. 6:**
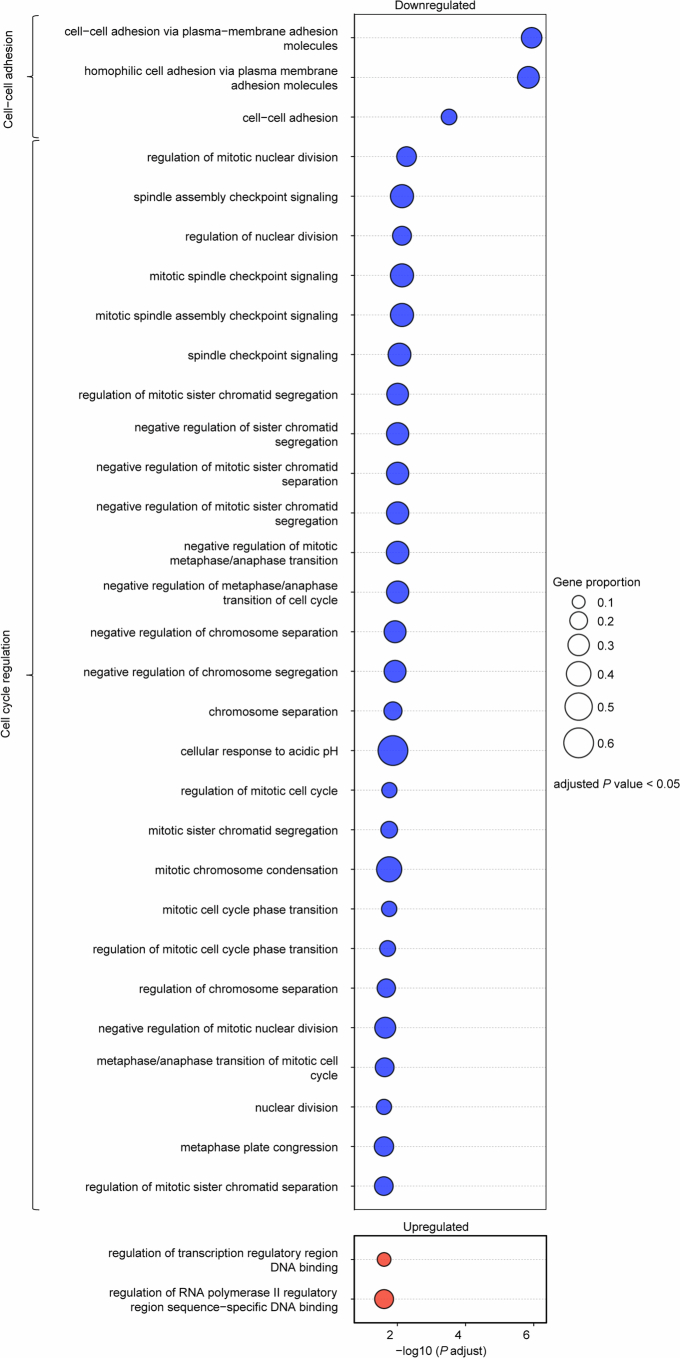
Pathway analysis in CTCs upon treatment with digoxin. Top 30 enriched pathways (over-representation analysis adjusted *P* value < 0.05) among downregulated (*top*) and upregulated (*bottom*) genes in circulating tumor cells (CTCs) deriving from blood samples post-digoxin intake.

We previously demonstrated that the dissociation of CTC clusters in patient-derived cell lines and xenografts causes a downregulation of stemness- and cell-cycle-related genes, alongside cell–cell adhesion disruption^11^. Here, we aimed to track changes in gene expression patterns in CTCs freshly isolated from an index patient over time, before and after treatment with digoxin, to confirm our preclinical observations. To this end, upon CTC enrichment and enumeration, we performed RNA sequencing (RNA-seq) of serial CTC samples from patient 5, unique for this purpose given the availability of multiple pretreatment samples (up to months before digoxin administration) and long treatment pauses in between various treatments (including digoxin), allowing longitudinal gene expression analysis with minimal likelihood for confounding factors. Blood samples were obtained at three time points before initiation of digoxin treatment (day −86, day −17 and day 0 pretreatment), and one time point after digoxin treatment (day 32), before the next line of systemic anticancer treatment (Extended Data Fig. 5a). Samples were collected and processed for next-generation RNA-seq as described previously^13^ (Extended Data Fig. 5b). Postdigoxin CTCs were characterized by differential expression of 708 genes compared with predigoxin samples, of which 685 were downregulated and 23 were upregulated (Extended Data Fig. 5c and Supplementary Tables 1 and 2). Strikingly, pathway analysis showed highly significant downregulation of cell-cycle-related genes (G2/M transition and E2F targets, for example, *CCNB2*, *PLK1*, *CHECK2* and *CDC25*; *P* < 0.05) and cell–cell adhesion molecules (for example, *PCDH12*, *CHD4* and *CHD24*; *P* < 0.05) compared with CTCs deriving from blood samples obtained before digoxin intake (Extended Data Fig. 5d, with the top 30 ontologies shown in Extended Data Fig. 6). This observation is highly consistent with our preclinical discoveries using patient-derived xenografts^11^, confirming cell–cell adhesion disruption and interference with cell cycle upon inhibition of the Na^+^/K^+^ ATPase.

In conclusion, the DICCT trial successfully demonstrated that a partial dissolution of CTC clusters can be achieved through the inhibition of the Na^+^/K^+^ ATPase in patients with metastatic breast cancer. We observed a similar dissolution effect in both homotypic and heterotypic clusters, along with a marked downregulation of genes involved in cell cycle regulation and cell–cell adhesion. Although clinical outcome endpoints were not assessed in this proof-of-concept study, the DICCT trial provides first-in-class evidence that supports the design of novel approaches for metastasis prevention. Of note, CTC clusters were observed in both lobular and ductal breast cancer subtypes. This finding is intriguing given the nature of lobular carcinomas, often characterized by E-cadherin loss^14,15^, yet compatible with previous knowledge of cell–cell adhesion components involved in the maintenance of CTC clusters^8,9,11^. While our study met its primary endpoint, we recognize some limitations and opportunities to improve future study designs. For instance, we observed a generally low number of CTCs along with intra- and interpatient variability in peripheral blood samples, negatively affecting the statistical power to detect changes. While our group has recently demonstrated a striking effect of circadian rhythmicity dictating CTC generation dynamics and suggesting highest CTC intravasation rates during sleep^16^, this study was conducted by sampling relatively small volumes of peripheral blood during morning hours. We envision that a tightly controlled, time-of-day-guided sampling (for example, night sampling in hospitalized patients) and/or sampling of larger volumes of blood (for example, through apheresis)^17^ could substantially reduce sampling error. Lastly, the effect of digoxin at a relatively low (maintenance) dose on cluster size was significant but mild. We highly anticipate future studies in this context, designed for a longer treatment duration, more frequent monitoring of drug serum levels or higher dosage or using refined Na^+^/K^+^ ATPase inhibitors with stronger cluster-dissolution activity and aimed at measuring clinical endpoints related to new metastasis development.

## Methods

### Inclusion and ethics

The study was approved by the Swiss authorities (BASEC-Nr. 2019-00673, BASEC-Nr. 2021-01939 and BASEC-Nr. 2020-00014) and in compliance with the Declaration of Helsinki.

### Study design and participants

DICCT (NCT03928210; DICCT/Swiss-GO-07) is a multicenter, investigator-initiated, prospective, single-arm, therapeutic exploratory phase 1 trial that was conducted in the University Hospital Basel, Cancer Center Baselland (Kantonsspital Baselland), and the Department of Gynaecology, University Hospital Zurich, in Switzerland. All patients (including both treated and control cohorts) were enrolled between July 2020 and July 2024. Patients aged 18 years or older with locoregionally recurrent or metastatic breast cancer with progressive disease not amenable to treatments with curative intent were eligible for study inclusion. Patients on concurrent treatment with digoxin or digitoxin, with inadequate renal, liver and marrow function, preexisting cardiac arrhythmias, an electrocardiogram (ECG) suggestive of or known hypertrophic cardiomyopathy, electrolyte disturbances, pregnancy, breastfeeding or a desire for childbearing and acute toxic effects of prior anticancer therapy Common Terminology Criteria for Adverse Events (CTCAE) version 4.0 grade >1 were excluded. Written informed consent was obtained from all participants. The clinical study protocol is provided in Supplementary Data 1. All blood specimens were obtained under the study protocol (BASEC-Nr. 2019-00673, BASEC-Nr. 2021-01939 and BASEC-Nr. 2020-00014) approved by the Swiss authorities (Cantonal Ethics Committee Basel, Cantonal Ethics Committee Zurich). Blood samples for CTC and cluster enumeration were obtained before any subsequent line of systemic therapy. In the presence of at least one homotypic or heterotypic CTC cluster, patients were enrolled into the treatment phase. Digoxin is authorized in Switzerland for treatment of heart failure and cardiac arrythmias, and target serum levels of digoxin in this trial were in accordance with the drug label. Patients received a daily maintenance dose of digoxin (0.125 mg or 0.25 mg pills, given before 10:00) for 7 days, calculated according to the renal function and the target serum digoxin concentration of 0.7 ng ml^−1^. Digoxin serum levels were measured on days 0 and 7. Single and cluster CTC enumeration were performed on day 0 (before and 2 h after digoxin intake), day 3 and day 7 (blood volume analyzed ranged from 5.5 to 20.8 ml). A total of 58 patients were screened, of whom 11 (18.9% of patients) were enrolled into the treatment phase and received digoxin (intention-to-treat population). Among these, two patients were excluded from the study: one because of the inability to reach the target serum level and another because of a digoxin-unrelated adverse event. Nonrandomized control untreated patients were enrolled under study protocol nos. 2021-01939 (approved by the *Kantonale Ethikkommission Kanton Zürich*) and 2020-00014 (approved by the *Ethikkommission Nordwest- und Zentralschweiz*). By applying the very same inclusion criteria as in our study, measurements were conducted at days 0, 3 and 7, and the very same calculations and considerations were applied as in our digoxin-treated cohort.

### CTC capture and CTC enumeration

Blood samples were obtained via peripheral venipuncture in EDTA vacutainers and processed for microfluidic-based CTC capture within 4 h from blood draw. Using the Parsortix Cell Separation System (ANGLE), CTCs were captured in Cell Separation cassettes (GEN3D6.5) and stained with an antibody cocktail containing anti-human EpCAM–AF488 (1:50; Cell Signaling Technology, CST5198), HER2–AF488 (1:50; BioLegend, 324410), EGFR–FITC (1:25; GeneTex, GTX11400) and CD45-AF647 (1:25; BioLegend, 304018). The number of captured CTCs, including single CTCs, CTC clusters and CTC–white blood cell (WBC) clusters, was determined while cells were still in the cassette. CTCs were then released from the cassette in phosphate-buffered saline (Gibco, 14190169) onto ultralow-attachment plates (Corning, 3471-COR) for downstream analysis.

### Statistical analysis

The estimated number of patients to be screened was between 50 and 60 patients, with an estimated 25% of blood samples harboring CTC clusters. Based on this, the expected number of patients with a digoxin serum level within the target range after treatment was 9 (80%), providing a power of 0.8 to estimate a mean treatment effect of digoxin of 1.1 (average CTC cluster size reduction, expressed in number of cells). The cumulative effect of digoxin treatment from baseline to day 7 was analyzed using a linear mixed model for repeated measures. The model included the covariate treatment time point as the fixed effect, and random intercept and a random slope for patient variables. In analyses using normalized variables and fold changes in log_2_ scale, infinite values were converted into the 0.5 + maximum noninfinite value. The CTC cluster proportion was defined as the ratio of CTC clusters detected over total CTC events. The association of digoxin levels and the average CTC cluster size normalized by baseline levels was evaluated using linear regression. For visualization, values were normalized using the average between the screening and day 0 predose (baseline).

### Direct metastatic potential assay

The 4T1 mouse mammary carcinoma cell line (derived from a female mouse) was obtained from the American Type Culture Collection (ATCC, CRL-2539). The cells were cultured in Dulbecco’s modified Eagle medium/nutrient mixture F-12 (DMEM/F-12, ThermoFisher, 11320033), supplemented with 10% fetal bovine serum (Gibco, 10500064) and antibiotic/antimycotic (Gibco, 15240062), and maintained in a humidified incubator at 37 °C with 20% O_2_ and 5% CO_2_ for a brief period of time before injection. The cell line was confirmed negative for mycoplasma contamination, and it does not belong to the commonly misidentified cell lines. All mouse experiments followed institutional and cantonal guidelines (protocol number 36338; approved by the *Kanton Zürich Veterinäramt*). Maximal permitted tumor size (2,800 mm^3^) was never exceeded. The animals were housed in a controlled environment with a room temperature maintained at 22 ± 2 °C and relative humidity at 55 ± 10%. The light–dark cycle was standardized to a 12-h photoperiod (12 h light, 12 h dark). Sample sizes were determined while adhering to the 3R principles (replacement, reduction and refinement) without predetermined calculations, but our sample sizes are similar to those reported in previous publications^8,16^. Mice were randomized (without blinding) before each experiment. A total of 2.5 × 10^5^ 4T1–red fluorescent protein (RFP)–luciferase cells were injected into the mammary fat pad of 8–12-week-old NSG female mice purchased from Charles River Laboratory. After 3.5 weeks of tumor development, blood was collected at 10:00 through terminal heart puncture. CTCs were captured using the Parsortix system (ANGLE) with the Cell Separation cassettes (GEN3D6.5) and stained with anti-mouse CD45-AF647 (1:50; clone 30-F11, BioLegend 103124) to distinguish CTCs from WBCs. Following capture, CTCs were released onto ultralow-attachment plates (Corning, 3471-COR) in 2 ml of phosphate-buffered saline (Gibco, 14190169). A total of 12 cells were individually picked from clusters of sizes 2, 3, 4, 6 and 12 using the CellCelector (Sartorius). Each size category of CTCs was injected into the tail vein of 6–8-week-old, tumor-free NSG recipient mice. The metastatic potential of the injected CTCs was monitored using in vivo imaging bioluminescence system. No animals or data points were excluded from the analysis.

### RNA-seq

Single CTCs, CTC clusters and CTC–WBC clusters were pooled into tubes for molecular characterization using next-generation sequencing. Using CellCelector, an automated single-cell picking system (Sartorius), pools of single CTCs, CTC clusters and CTC–WBC clusters (range of 5–40 cells) were collected and immediately transferred into tubes (Axygen, 321-032-501, Thermofisher AB-0620) containing 10 µl RLT Plus lysis buffer and 1 U SUPERase IN RNase inhibitor (Invitrogen, AM2694). Samples were immediately frozen and kept at −80 °C until further processing. Following a previously published protocol for parallel DNA and RNA sequencing from individual cells^13^, genomes and transcriptomes of lysed cells were separated. Amplified cDNA was prepared on-bead according to the Smart-seq2 protocol. Libraries were prepared using Nextera XT (Illumina) and sequenced on an Illumina NextSeq2000 instrument in 101-bp single-read mode. This yielded a median raw sequencing depth of 7.8 million reads per sample.

### RNA-seq analysis

Sequencing reads were quality trimmed with Trim Galore! (v0.6.6, https://www.bioinformatics.babraham.ac.uk/projects/trim_galore/; parameters: --q 20 --length 20. Quality assessment of RNA-seq data was carried out using FastQC (v0.11.9, https://www.bioinformatics.babraham.ac.uk/projects/fastqc) and FastQ Screen (v0.15.2, https://www.bioinformatics.babraham.ac.uk/projects/fastq_screen/) and visualized with MultiQC (v1.9). Trimmed reads were aligned to human (GRCh38) genome reference using STAR (v.2.7.9a) with splice junctions from the human GENCODE annotation (release 40). Resulting BAM files were sorted by Samtools (v1.10), and the alignment quality was evaluated using RSeQC (v.4.0.0). The gene-level expression counts were computed with featureCounts from the subread package (v.2.0.3; parameters: -t exon -g gene_id--minOverlap 10 -Q 10) using the human gene annotations from GENCODE (release 40). Samples were retained for further analyses if they had at least 500,000 reads, at least 5,000 genes with nonzero expression and less than 50% of reads mapping to mitochondrial genes.

### Differential gene expression

Differential expression was computed using DESeq2 R/Bioconductor package (v1.38.3) using the Wald test for significance. Before differential expression analysis, genes detected in less than 80% of *n* samples, *n* being the size of the smallest group, were removed from the analysis. *P* values were adjusted for multiple comparisons using the Benjamini–Hochberg method. Functional enrichment analysis querying Biological Processes from Gene Ontology (GO) was conducted using the function enrichGO implemented in the R/Bioconductor package clusterProfiler (v4.6.0). We selected genes with an adjusted *P* value ≤0.1 as input for the functional analysis. We removed redundant enriched GO terms using semantic similarity with a cutoff of 0.6 as implemented in the function simplify from the R/Bioconductor package clusterProfiler (v4.6.0).

### Data analysis

Data analysis, statistical testing and visualization were conducted in R (version 4.2.2; R Foundation for Statistical Computing) Bioconductor (v.3.16), GraphPad Prism (v 9.0.2) and BioRender.com.

### Reporting summary

Further information on research design is available in the Nature Portfolio Reporting Summary linked to this article.

## Online content

Any methods, additional references, Nature Portfolio reporting summaries, source data, extended data, supplementary information, acknowledgements, peer review information; details of author contributions and competing interests; and statements of data and code availability are available at 10.1038/s41591-024-03486-6.

## Supplementary information

Supplementary Information Supplementary Tables 1 and 2 and Data 1.

Reporting Summary

## Source data

Source Data Fig. 1 Source data for Extended Fig. 4.

## Figures and Tables

**Extended Data Table 1:**
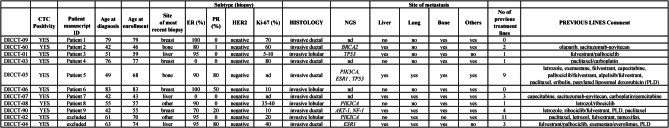
Clinical characteristics of digoxin treated breast cancer patients

Table showing the age (years) at primary diagnosis, age (years) at CTC enumeration, subtype of most recent biopsy (% of ER-positive cells, % of PR-positive cells, % of Ki67-positive cells, HER2 amplification status, histologic subtype (ductal, lobular), next-generation sequencing (NGS), site of metastasis, number and type of previous systemic treatment lines. nd: not determined.

**Extended Data Table 2:**
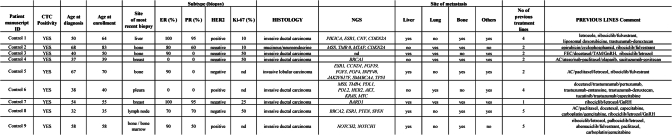
Clinical characteristics of untreated, control breast cancer patients

Table showing the age (years) at primary diagnosis, age (years) at CTC enumeration, subtype of most recent biopsy (% of ER-positive cells, % of PR-positive cells, % of Ki67-positive cells, HER2 amplification status, histologic subtype (ductal, lobular, mucinous/neuroendocrine), next-generation sequencing (NGS), site of metastasis, number and type of previous systemic treatment lines. nd: not determined.

**Extended Data Table 3:**
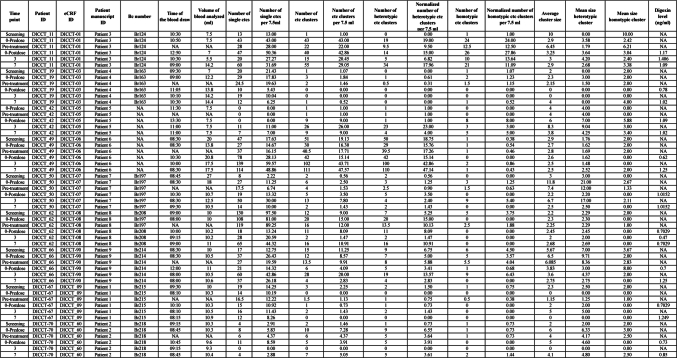
Circulating tumor cells enumeration, composition, and digoxin serum levels in the treated patients

Table describing the volume of blood analyzed, the total number of single CTCs, the total number of CTC clusters, the number of homotypic CTC clusters and the number of heterotypic CTC-WBC clusters per 7.5 ml of peripheral blood, the average cluster size and digoxin serum levels. Pre-treatment value is the calculated average of Screening and 0-pre-dose values, when both were available. NA: not available.

**Extended Data Table 4:**
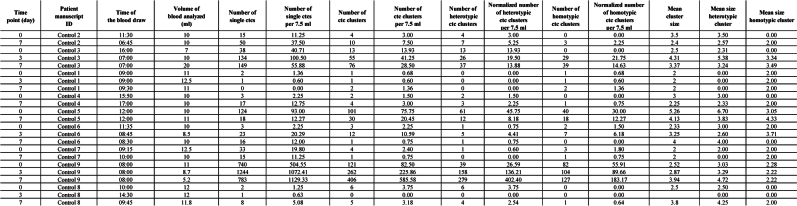
Circulating tumor cells enumeration and composition in the untreated control patients

Table describing the volume of blood analyzed, the total number of single CTCs, the total number of CTC clusters, the number of homotypic CTC clusters and the number of heterotypic CTC-WBC clusters per 7.5 ml of peripheral blood, and the average cluster size.

## Data Availability

RNA-seq data have been deposited in the Gene Expression Omnibus (GEO, National Center for Biotechnology Information; accession number GSE249233). Processed transcriptomics data, large datasets and other files required for reproducibility are available via Zenodo at 10.5281/zenodo.10215049 (ref. ^18^). The human reference genome (GRCh38) human gene annotation (release 40) was downloaded from GENCODE (https://www.gencodegenes.org). Source data are provided with this paper.

## Code availability

Code related to the RNA-seq analysis of this study, together with the description of how to reproduce the analysis workflow, is available via GitHub at https://github.com/TheAcetoLab/dicct-trial. The link is public.
