# Supplementary material for: Digoxin for reduction of circulating tumor cell cluster size in metastatic breast cancer: a proof-of-concept trial
Source: Nat Med. 2025 Jan 24;31(4):1120–4. doi: 10.1038/s41591-024-03486-6 (PMC12003195; doi:10.1038/s41591-024-03486-6)
Supplement: Supplementary file 1 — Supplementary Tables 1 and 2 and Data 1. [file 41591_2024_3486_MOESM1_ESM.pdf]

nature medicine

Supplementary information

https://doi.org/10.1038/s41591-024-03486-6

Digoxin for reduction of circulating tumor cell cluster size in metastatic breast cancer: a proof-of-concept trial

In the format provided by the authors and unedited

Supplementary Table 1: Genes downregulated in CTCs post-digoxin.

Table listing the genes downregulated in CTC pools obtained 32 days post-digoxin intake (n = 2) compared to CTC pools obtained at 86 days, 17 days and zero days prior to digoxin intake (n = 5) in patient 5. Gene ID, fold change, P value, and adjusted P-value are shown (edgeR likelihood ratio test for differential expression,

Gene ID	Log2FoldChang	Base Mean	*P* value	Adjusted *p* val	Direction
KCNG4	-24.65	46.51	1.55E-09	2.71E-11	Down
KIF18B	-24.57	43.37	1.77E-09	0.000000282	Down
PARVG	-24.45	40.64	2.1E-09	0.000000773	Down
GRM4	-24.36	37.53	2.41E-09	0.000000773	Down
IQCN	-12.81	193.87	3.48E-09	0.00000202	Down
ESPL1	-12.67	177.06	5.3E-11	0.00000202	Down
RRM2	-12.18	125.55	3.26E-09	0.00000202	Down
LRP8	-12.16	124.10	1.61E-08	0.00000202	Down
RADIL	-11.94	106.94	0.000000242	0.00000202	Down
RBPJL	-11.84	99.21	0.000000043	0.00000202	Down
TIGD1	-11.79	96.42	0.000000311	0.00000203	Down
C13orf46	-11.70	90.33	0.000000122	0.00000214	Down
ENSG0000028	-11.66	87.98	0.000000212	0.00000265	Down
PDXP	-11.61	85.06	0.00000014	0.00000265	Down
DNHD1	-11.54	81.29	0.000000391	0.00000286	Down
ZFHX2	-11.46	76.20	0.000955	0.00000373	Down
AGFG2	-11.37	72.15	0.000000279	0.00000373	Down
SLCO2A1	-11.35	70.95	0.000000927	0.00000528	Down
ZEB2	-11.31	69.02	0.000000157	0.00000721	Down
EID3	-11.21	63.72	3.23E-08	0.0000078	Down
E2F2	-11.00	55.26	0.00319	0.00000816	Down
FRMD5	-10.87	51.08	0.000000339	0.00000836	Down
TMEM201	-10.81	48.90	0.000000349	0.0000114	Down
RGS3	-10.79	48.28	0.00000146	0.0000127	Down
SH2B3	-10.78	48.12	0.00000047	0.0000132	Down
AMER1	-10.75	46.84	0.00000543	0.0000132	Down
NEMP1	-10.74	45.67	3.49E-08	0.0000133	Down
ITGB2	-10.68	44.55	0.000000662	0.0000133	Down
WTIP	-10.60	42.46	0.00000997	0.0000153	Down
MDC1	-10.58	147.91	2.9E-10	0.0000153	Down
SPOCK1	-10.57	41.56	0.000006	0.0000155	Down
DLEU7	-10.52	39.49	0.000000304	0.0000194	Down
CCL3	-10.48	38.67	0.00000417	0.0000194	Down
ALOXE3	-10.33	80.57	0.000000676	0.0000194	Down
EML6	-10.33	35.06	0.00000742	0.0000202	Down
PMEL	-10.33	35.08	0.0000337	0.0000256	Down
PLA2G4B	-10.32	35.10	0.000000829	0.0000327	Down
KIF26A	-10.25	33.23	0.00000968	0.0000327	Down
PDE9A	-10.24	32.94	0.000019	0.0000333	Down
ZNF169	-10.23	32.77	0.0000212	0.0000365	Down
IQCC	-10.22	32.59	0.00000831	0.0000365	Down
NKD2	-10.21	31.97	0.00000441	0.0000397	Down

SPATC1	-10.21	32.28	0.0000506	0.0000415	Down
CACNB1	-10.20	137.26	0.00000321	0.0000512	Down
PLXDC1	-10.16	31.29	0.0000166	0.0000513	Down
LIMK1	-10.14	30.69	0.0000406	0.0000519	Down
CLIC5	-10.12	30.44	0.00000544	0.0000549	Down
PTCH1	-10.11	30.22	0.0000525	0.0000579	Down
PCBP3	-10.08	29.52	0.000084	0.0000606	Down
CCL3L1	-10.05	112.42	0.000000407	0.0000637	Down
ENDOV	-10.05	28.95	0.00000396	0.0000637	Down
PRDM11	-10.03	28.51	0.0000957	0.0000637	Down
TNNT2	-10.00	27.90	0.0000604	0.0000681	Down
LCP2	-9.98	27.35	0.0000461	0.0000689	Down
FRMPD3	-9.97	27.22	0.0000104	0.0000706	Down
HERC3	-9.96	27.30	0.00000455	0.0000744	Down
RIPOR3	-9.95	26.77	0.0000391	0.0000761	Down
SKA3	-9.92	25.96	0.00000323	0.0000799	Down
MSANTD1	-9.90	26.18	0.0000199	0.0000813	Down
KLHL29	-9.88	25.71	0.00005	0.0000813	Down
DNAH7	-9.86	25.27	0.00000402	0.0000813	Down
CDH24	-9.86	25.30	0.00000509	0.0000813	Down
SH3RF2	-9.86	25.29	0.0000693	0.0000881	Down
KCNB1	-9.82	24.66	0.000079	0.0000896	Down
AMIGO3	-9.81	24.39	0.00000412	0.0000967	Down
PLK4	-9.81	24.25	0.0000145	0.000103	Down
ADAMTSL4	-9.79	333.08	2.54E-15	0.000104	Down
ANO7	-9.79	55.21	5.83E-08	0.000104	Down
ENSG00000287	-9.79	24.23	0.00000791	0.000104	Down
AOAH	-9.73	22.92	0.0000845	0.000108	Down
SHH	-9.69	22.54	0.000109	0.000113	Down
KCNQ4	-9.68	22.40	0.0000173	0.000123	Down
RAB44	-9.67	22.21	0.0000232	0.000123	Down
KIF4A	-9.66	21.76	0.00000824	0.000131	Down
ICAM5	-9.66	21.67	0.000053	0.000132	Down
MYBL2	-9.65	150.53	4.51E-08	0.000176	Down
CTPS1	-9.65	50.27	0.000000909	0.000176	Down
ORC1	-9.65	21.72	0.0000577	0.000179	Down
UCP3	-9.65	22.00	0.000232	0.00018	Down
CHGA	-9.64	21.59	0.0000624	0.000184	Down
PTGS1	-9.64	21.86	0.000859	0.000185	Down
PLEKHH2	-9.63	21.62	0.0000131	0.000188	Down
NXN	-9.63	21.74	0.000196	0.000188	Down
TRAIP	-9.63	21.72	0.000375	0.000203	Down
PGAP1	-9.62	21.54	0.000179	0.000204	Down
GPR84	-9.62	21.46	0.000943	0.00027	Down
DNAH12	-9.61	21.37	0.000188	0.000298	Down
ITGA2B	-9.61	21.39	0.000194	0.000318	Down
CAND2	-9.61	21.39	0.000199	0.000344	Down
SUFU	-9.59	21.16	0.0000229	0.000379	Down
ABCD1	-9.58	21.03	0.00000609	0.000379	Down
MPIG6B	-9.58	20.95	0.000952	0.00043	Down

COLQ	-9.57	20.78	0.000238	0.000454	Down
SNX30	-9.54	46.73	0.00000371	0.000455	Down
AURKB	-9.53	20.24	0.0000231	0.000455	Down
CYTH3	-9.53	20.23	0.000181	0.000457	Down
H3C2	-9.52	19.58	0.000129	0.000458	Down
ANKRD30B	-9.49	19.37	0.00000857	0.000469	Down
PLEKHF1	-9.49	19.74	0.0000804	0.000469	Down
BTBD19	-9.46	19.38	0.0000723	0.000469	Down
VASH2	-9.46	19.21	0.001	0.00048	Down
CD160	-9.45	19.05	0.0000235	0.000483	Down
CDK3	-9.43	18.85	0.000057	0.000493	Down
KLHL3	-9.43	18.94	0.000231	0.000521	Down
KNG1	-9.43	18.82	0.00116	0.000531	Down
IFNLR1	-9.42	42.95	0.000000306	0.000532	Down
SLC16A8	-9.40	18.56	0.0000241	0.000532	Down
PHF19	-9.38	17.81	0.0000475	0.000532	Down
NPAS2	-9.38	18.19	0.000514	0.000532	Down
DCST1	-9.37	18.04	0.0000416	0.000542	Down
HAP1	-9.36	62.67	0.00000462	0.000554	Down
NTRK3	-9.35	17.90	0.00058	0.00057	Down
C1orf116	-9.35	17.87	0.00122	0.000574	Down
MYO15B	-9.33	146.68	1.89E-09	0.00067	Down
C20orf144	-9.33	17.70	0.0000888	0.000676	Down
C22orf23	-9.33	17.65	0.00146	0.000676	Down
GCNA	-9.32	17.20	0.000124	0.000676	Down
LRRC25	-9.32	17.60	0.000309	0.000714	Down
CD300E	-9.30	17.26	0.000591	0.000731	Down
FNDC11	-9.30	17.29	0.00143	0.000731	Down
AGAP2	-9.30	17.25	0.00152	0.000731	Down
ZNF695	-9.29	17.01	0.00000536	0.000734	Down
MCM10	-9.27	16.61	0.000306	0.000736	Down
OSR2	-9.25	16.69	0.0000696	0.000736	Down
ZDBF2	-9.25	16.57	0.000551	0.000739	Down
HEY1	-9.24	16.47	0.000276	0.000747	Down
VLDLR	-9.23	16.39	0.000163	0.000758	Down
STRIP2	-9.23	16.51	0.000364	0.00077	Down
PCDH12	-9.23	16.51	0.000709	0.000799	Down
GPR176	-9.23	16.25	0.00141	0.000813	Down
POLQ	-9.22	15.98	0.000194	0.000813	Down
PCDHB15	-9.22	16.22	0.000217	0.000833	Down
TLCD3A	-9.21	15.93	0.000123	0.000833	Down
BRIP1	-9.21	16.01	0.000202	0.000885	Down
SLC23A1	-9.20	16.16	0.0000136	0.000896	Down
SLC43A2	-9.20	15.93	0.0000239	0.00102	Down
FGFR4	-9.20	36.86	0.000124	0.00102	Down
SLC7A9	-9.18	15.90	0.00193	0.00103	Down
NLRP3	-9.17	15.88	0.000218	0.00104	Down
ORC6	-9.16	15.52	0.000318	0.00109	Down
KIF14	-9.14	15.19	0.0000856	0.00109	Down
ADAM32	-9.13	15.27	0.0000292	0.00113	Down

CCDC116	-9.13	15.29	0.000328	0.00113	Down
RNASE10	-9.12	15.17	0.00191	0.0012	Down
S1PR5	-9.10	15.03	0.00205	0.0012	Down
ALAS2	-9.09	14.98	0.000845	0.00121	Down
C4orf54	-9.08	14.82	0.00235	0.00125	Down
ZNF367	-9.06	14.28	0.000112	0.00136	Down
SPIN4	-9.06	14.18	0.000125	0.00142	Down
EOGT	-9.05	14.32	0.0000694	0.00142	Down
CNNM1	-9.04	14.40	0.00208	0.00149	Down
HTR6	-9.01	14.11	0.00237	0.00158	Down
ACAP1	-8.98	13.78	0.0000846	0.00158	Down
PCDHB13	-8.98	13.89	0.0000925	0.00158	Down
ANO2	-8.96	13.71	0.000855	0.00158	Down
GUCY1A2	-8.94	13.38	0.00251	0.00158	Down
VSX1	-8.94	13.46	0.00286	0.00158	Down
TONSL	-8.93	62.29	0.00000126	0.00158	Down
PTPRN	-8.91	13.20	0.00278	0.0016	Down
LZTR1	-8.90	133.91	0.000000435	0.00161	Down
PAPLN	-8.89	46.08	0.000000138	0.00164	Down
XIRP1	-8.88	12.83	0.0027	0.0017	Down
UPK3B	-8.85	12.66	0.000811	0.0018	Down
HMCN1	-8.85	12.61	0.00284	0.00181	Down
KIFC1	-8.84	12.36	0.000108	0.00181	Down
PTPN21	-8.83	12.40	0.0000975	0.00181	Down
ZNF696	-8.82	12.47	0.00142	0.00186	Down
ESR2	-8.82	12.36	0.00335	0.00186	Down
ENSG0000025ᵴ	-8.78	12.04	0.000747	0.00196	Down
CELF6	-8.78	12.07	0.00123	0.00197	Down
CENPM	-8.77	11.74	0.000601	0.00199	Down
IL17RB	-8.76	11.95	0.0000283	0.00205	Down
CHST6	-8.73	40.24	0.000152	0.00207	Down
RASAL1	-8.72	11.55	0.000188	0.00207	Down
GRIK3	-8.71	40.98	6.16E-08	0.00216	Down
CILP2	-8.71	11.02	0.0000631	0.0022	Down
ZMYND15	-8.70	25.97	0.00000127	0.0023	Down
ARHGAP33	-8.69	52.15	0.00000138	0.00233	Down
LPAR1	-8.69	11.05	0.000709	0.00233	Down
FUT11	-8.68	11.22	0.000467	0.00239	Down
CELF4	-8.68	11.29	0.00123	0.00239	Down
TSPAN10	-8.67	11.13	0.00104	0.00239	Down
BUB1B	-8.66	11.03	0.00103	0.00239	Down
TBCEL	-8.65	11.01	0.000244	0.00239	Down
ALG12	-8.64	66.07	0.00000717	0.00239	Down
LBX2	-8.64	10.88	0.000151	0.00243	Down
RSKR	-8.64	10.97	0.00217	0.00243	Down
TP53TG5	-8.63	10.93	0.000656	0.00244	Down
NDC80	-8.63	10.78	0.000848	0.0025	Down
CEP152	-8.62	10.75	0.000232	0.00256	Down
CIT	-8.59	61.61	0.000000464	0.00257	Down
EPOP	-8.59	10.41	0.000717	0.00264	Down

GINS1	-8.58	10.31	0.000186	0.00276	Down
FANCG	-8.55	23.41	0.0000792	0.00276	Down
PHF13	-8.52	9.98	0.000213	0.00276	Down
ENSG0000026[illegible]	-8.52	10.03	0.00155	0.00284	Down
ENSG0000026[illegible]	-8.51	22.58	0.00000541	0.00284	Down
CDH4	-8.50	22.81	0.00124	0.00285	Down
H3C10	-8.50	9.70	0.00169	0.00294	Down
MCM2	-8.49	35.18	0.000000117	0.00302	Down
EFNA2	-8.49	9.60	0.00108	0.00302	Down
ENSG0000026[illegible]	-8.46	9.69	0.00055	0.00304	Down
CDC25C	-8.45	22.05	0.0000632	0.00314	Down
PCSK4	-8.43	9.38	0.000115	0.00315	Down
SCNN1D	-8.42	65.54	0.00000059	0.00316	Down
POLD1	-8.42	63.91	0.00000243	0.00317	Down
ENSG0000026[illegible]	-8.42	9.16	0.00127	0.00317	Down
VXN	-8.41	9.15	0.000291	0.0032	Down
SULF1	-8.40	97.64	8.66E-08	0.00321	Down
E2F7	-8.40	8.96	0.0016	0.00321	Down
ENSG0000025[illegible]	-8.39	21.05	0.000000844	0.00323	Down
IL10	-8.37	8.81	0.00166	0.00328	Down
IFIT3	-8.36	31.12	0.0000232	0.00328	Down
RAPGEF3	-8.31	93.74	0.00000163	0.0033	Down
SYT9	-8.31	8.51	0.00102	0.00333	Down
KHDC1	-8.29	8.50	0.00222	0.00333	Down
ENSG0000024[illegible]	-8.27	19.14	0.0000337	0.00333	Down
BTN3A3	-8.27	8.25	0.00295	0.00333	Down
MMS22L	-8.26	8.52	0.000316	0.00334	Down
OGN	-8.26	8.18	0.00285	0.00334	Down
ASTL	-8.24	29.11	0.00000831	0.00334	Down
TMEM88	-8.24	8.20	0.0022	0.00334	Down
ST3GAL5	-8.22	45.00	0.00034	0.00345	Down
CDCA5	-8.21	36.79	0.0000681	0.00355	Down
ERF	-8.20	58.74	2.87E-08	0.0036	Down
TAF4	-8.20	93.88	0.000000224	0.0037	Down
SLC10A1	-8.20	7.87	0.00224	0.0037	Down
PIGW	-8.18	7.57	0.00132	0.0037	Down
H1-5	-8.16	7.53	0.00264	0.00374	Down
FUT10	-8.15	7.72	0.000632	0.00385	Down
HS6ST2	-8.10	17.20	0.000726	0.00386	Down
H2BC11	-8.10	7.32	0.000899	0.00386	Down
SOWAHA	-8.10	7.58	0.00163	0.00386	Down
FAM111B	-8.10	7.40	0.00298	0.00386	Down
C1orf100	-8.07	7.20	0.00332	0.00386	Down
PLB1	-8.06	25.32	0.00073	0.00386	Down
HUNK	-8.05	41.80	0.000013	0.00387	Down
RAVER1	-8.04	80.47	1.41E-09	0.00397	Down
EVI5L	-8.03	31.30	0.000351	0.00412	Down
LARGE2	-8.03	24.84	0.000398	0.00423	Down
POTEC	-7.97	15.91	0.00275	0.00426	Down
ALG9	-7.94	15.03	0.000104	0.00426	Down

HSPA2	-7.94	15.44	0.00043	0.00426	Down
NFATC1	-7.93	68.84	0.00000262	0.00427	Down
DNAH1	-7.91	156.49	8.93E-09	0.0043	Down
C1QTNF6	-7.91	58.48	0.0000664	0.00438	Down
CBLN3	-7.91	6.65	0.00189	0.00448	Down
CENPQ	-7.86	6.18	0.0016	0.00458	Down
SASH1	-7.82	28.15	0.000136	0.00458	Down
CHEK2	-7.79	14.06	0.000178	0.00458	Down
MOB3A	-7.73	20.64	0.0000151	0.0046	Down
HROB	-7.70	22.30	0.000112	0.0046	Down
AMT	-7.70	13.18	0.00127	0.00467	Down
ADAMTS10	-7.69	33.28	0.00001	0.00467	Down
FOSL1	-7.67	21.96	0.000153	0.00472	Down
PAX9	-7.62	18.95	0.000312	0.00472	Down
EEFSEC	-7.58	21.27	0.000233	0.00474	Down
ZDHHC11	-7.56	18.31	0.000141	0.00476	Down
ZNF257	-7.55	31.40	0.000116	0.0048	Down
FOXM1	-7.50	51.64	0.00000432	0.0048	Down
INPP5B	-7.49	34.51	0.00000903	0.00493	Down
IGHMBP2	-7.48	88.35	5.92E-09	0.00497	Down
LOXL1	-7.48	106.47	0.00000477	0.00497	Down
ABTB1	-7.47	47.15	0.00000046	0.00497	Down
ACAD10	-7.44	81.87	2.46E-08	0.00497	Down
CAPRIN2	-7.43	19.06	0.000294	0.00497	Down
ADPRHL1	-7.41	82.62	0.000041	0.00505	Down
LSM11	-7.41	16.16	0.00119	0.00508	Down
BEND3	-7.41	20.40	0.00132	0.00509	Down
MAPKBP1	-7.39	110.49	0.000000473	0.00512	Down
SPRED1	-7.39	16.42	0.0000615	0.00529	Down
PLPPR3	-7.37	20.92	0.000271	0.00531	Down
ADCK2	-7.36	30.11	0.000281	0.00532	Down
SMCHD1	-7.33	130.36	1.09E-09	0.00544	Down
ZC2HC1C	-7.32	34.47	0.000118	0.00551	Down
ADSS1	-7.30	84.31	0.00000133	0.0058	Down
SECTM1	-7.30	86.46	0.0000163	0.0058	Down
MTFP1	-7.30	9.99	0.00236	0.00581	Down
TICRR	-7.29	40.66	0.000865	0.00581	Down
CUL9	-7.27	96.09	0.00000064	0.00587	Down
POMT2	-7.25	28.81	0.0000314	0.0059	Down
TRPV1	-7.24	75.98	0.0000654	0.006	Down
HELZ2	-7.23	73.88	0.00000075	0.00611	Down
TK1	-7.23	49.33	0.000216	0.00623	Down
CCNB2	-7.21	9.35	0.00315	0.00623	Down
PFAS	-7.20	24.28	0.000137	0.0063	Down
NABP1	-7.17	63.28	0.00000578	0.00651	Down
LIN54	-7.17	26.53	0.0000695	0.00652	Down
NUF2	-7.15	13.19	0.00109	0.00654	Down
DMPK	-7.14	71.12	5.96E-09	0.00654	Down
PASK	-7.12	35.27	0.000043	0.00657	Down
GPT2	-7.09	32.59	0.000785	0.00657	Down

RNF166	-7.07	70.19	0.000000168	0.00657	Down
FAXC	-7.07	12.67	0.000692	0.00673	Down
MAP3K9	-7.05	85.28	2.9E-10	0.00676	Down
STXBP1	-7.01	20.13	0.000493	0.00676	Down
MEGF6	-7.00	84.46	0.00000436	0.00679	Down
USP49	-6.99	51.90	3.09E-08	0.00687	Down
ENSG00000265	-6.98	16.73	0.0000111	0.00687	Down
SPTBN2	-6.96	60.11	0.0000014	0.00687	Down
ARHGAP23	-6.96	50.06	0.000085	0.00691	Down
BIRC5	-6.94	27.05	0.0000853	0.00699	Down
PLEKHG4B	-6.93	25.97	0.000723	0.00705	Down
SLC25A27	-6.92	11.52	0.00259	0.00705	Down
NCAPD3	-6.89	76.98	0.0000114	0.00711	Down
FGD1	-6.85	39.13	0.000213	0.00711	Down
LRATD1	-6.85	20.76	0.00246	0.00731	Down
CLSTN3	-6.84	45.19	0.0000363	0.00731	Down
CDT1	-6.83	41.20	0.00000147	0.00731	Down
NCAPH	-6.83	14.59	0.000567	0.00734	Down
PTPRH	-6.83	12.65	0.000791	0.00734	Down
TRAF1	-6.82	21.34	0.0000973	0.00734	Down
TNFRSF19	-6.81	27.63	0.000232	0.00734	Down
MYO9B	-6.78	89.89	1.57E-09	0.00734	Down
GGT7	-6.77	17.16	0.00018	0.0074	Down
EPN3	-6.75	23.31	0.000261	0.00754	Down
MAP3K10	-6.75	6.75	0.00207	0.00757	Down
S100A1	-6.74	45.86	0.00000131	0.0076	Down
PKN3	-6.68	33.11	0.0000231	0.0076	Down
GPX3	-6.67	36.06	0.00000926	0.0076	Down
H3C12	-6.63	28.04	0.0000682	0.0076	Down
RELT	-6.62	45.80	0.000041	0.0076	Down
ZNF445	-6.60	55.07	0.000565	0.0076	Down
FSCN1	-6.60	9.12	0.00139	0.0076	Down
KNL1	-6.59	17.13	0.00171	0.0076	Down
DNM1	-6.54	64.91	0.00034	0.00764	Down
ZNF713	-6.53	15.04	0.000245	0.0077	Down
FSCN2	-6.51	21.70	0.00247	0.0077	Down
GHDC	-6.50	11.40	0.00293	0.00778	Down
KIFC3	-6.49	58.17	0.0000679	0.00778	Down
TROAP	-6.48	11.40	0.00308	0.00778	Down
TMEM250	-6.47	40.26	0.0000085	0.00778	Down
DRC3	-6.47	32.95	0.000364	0.00778	Down
PRR3	-6.45	26.17	0.000267	0.00792	Down
TRMT9B	-6.45	20.91	0.00218	0.00804	Down
PGS1	-6.39	89.15	0.0000443	0.0081	Down
SH3TC2	-6.38	33.69	0.000254	0.00815	Down
KMT5C	-6.37	35.93	0.0000143	0.00815	Down
ANLN	-6.36	144.03	0.0000861	0.00819	Down
CCNO	-6.36	18.04	0.000114	0.00819	Down
CCL4L2	-6.35	44.19	0.000519	0.00826	Down
GPR39	-6.30	23.28	0.00177	0.00829	Down

SCHIP1	-6.27	18.80	0.00246	0.00835	Down
CES4A	-6.26	28.49	0.000512	0.00836	Down
ZNF264	-6.25	49.90	0.0000505	0.00846	Down
SLC25A18	-6.23	41.31	0.000396	0.00858	Down
LSS	-6.22	113.93	0.000000365	0.00871	Down
RGP1	-6.20	123.76	0.00000883	0.00884	Down
PBK	-6.19	17.66	0.000974	0.00889	Down
LTB4R2	-6.18	26.59	0.0000134	0.00924	Down
MTA2	-6.18	55.28	0.000124	0.00929	Down
DNAJC4	-6.17	87.11	0.000448	0.00935	Down
PHETA1	-6.16	48.51	0.00013	0.00935	Down
LRRC3	-6.13	26.85	0.000257	0.00938	Down
HSPG2	-6.12	145.23	0.0000355	0.00938	Down
ZNF319	-6.12	63.11	0.00215	0.00938	Down
CCDC157	-6.11	26.83	0.000552	0.00938	Down
XPNPEP3	-6.11	14.33	0.000653	0.00938	Down
STK10	-6.10	66.66	0.000000062	0.00964	Down
RGS12	-6.09	100.22	1.73E-08	0.00967	Down
PLEKHA2	-6.09	29.44	0.00000406	0.0098	Down
MAML3	-6.09	14.81	0.00179	0.00987	Down
ZNF469	-6.08	88.25	0.000177	0.00987	Down
PEAR1	-6.08	75.54	0.000455	0.00987	Down
DGCR2	-6.05	40.33	0.000000261	0.0102	Down
RRP9	-6.05	24.16	0.0018	0.0102	Down
ADAD2	-6.04	50.01	0.00298	0.0103	Down
S100PBP	-6.01	19.75	0.000662	0.0103	Down
PLCD4	-5.98	11.51	0.0022	0.0103	Down
KIRREL3	-5.97	79.37	0.00026	0.0103	Down
LRP3	-5.97	20.64	0.00327	0.0103	Down
MAP3K6	-5.94	22.67	0.000128	0.0103	Down
PIP5K1C	-5.93	48.45	4.32E-08	0.0103	Down
FLVCR2	-5.93	54.25	0.000471	0.0104	Down
KIAA1549	-5.92	92.86	0.000000217	0.0106	Down
NDOR1	-5.91	43.56	0.000000675	0.0107	Down
LRRC45	-5.91	39.15	0.000421	0.0111	Down
CDC42BPG	-5.90	77.36	0.0000294	0.0111	Down
DOK7	-5.89	30.97	0.000791	0.0111	Down
H2BC18	-5.88	21.77	0.00036	0.0116	Down
ACSL1	-5.86	49.26	0.000202	0.0116	Down
H2AC17	-5.86	18.30	0.00232	0.0116	Down
ESCO2	-5.82	15.37	0.0016	0.0116	Down
TDP1	-5.81	24.24	0.000206	0.0118	Down
FRY	-5.80	30.67	0.0000427	0.0118	Down
HPS6	-5.78	26.80	0.00208	0.0118	Down
ZC3H7B	-5.75	91.05	0.0000787	0.0118	Down
SMCR8	-5.73	166.92	0.0000639	0.012	Down
BTBD2	-5.73	34.89	0.000114	0.0121	Down
PPIL6	-5.73	15.29	0.000244	0.0121	Down
CCDC149	-5.73	53.63	0.000982	0.0122	Down
FBXL16	-5.71	34.00	0.000355	0.0122	Down

GSAP	-5.71	32.01	0.000863	0.0122	Down
ENTPD7	-5.71	14.63	0.00283	0.0123	Down
STK11	-5.70	40.01	0.00163	0.0125	Down
ALDH5A1	-5.70	9.84	0.0029	0.0126	Down
CFAP92	-5.69	59.28	0.0000164	0.0126	Down
ITGAX	-5.69	51.76	0.00218	0.0129	Down
SYNPO	-5.67	75.08	0.0000259	0.0129	Down
SMARCB1	-5.65	152.24	0.0000656	0.013	Down
ENSG00000267	-5.63	13.96	0.00311	0.013	Down
TACC3	-5.61	101.21	0.0000549	0.0132	Down
REXO1	-5.59	38.87	0.000829	0.0133	Down
ZNF821	-5.59	15.18	0.00257	0.0133	Down
ZYG11A	-5.58	25.92	0.000286	0.0134	Down
SLC26A1	-5.57	69.78	0.000076	0.0134	Down
EXT2	-5.57	40.65	0.000418	0.0137	Down
FBXO46	-5.56	18.43	0.0000747	0.0139	Down
CRTC2	-5.55	75.75	0.00000524	0.014	Down
RIPOR1	-5.55	88.85	0.00000559	0.0141	Down
WNT4	-5.55	59.70	0.00114	0.0141	Down
LRRC8B	-5.54	23.80	0.000604	0.0141	Down
MFSD4B	-5.54	124.71	0.00105	0.0142	Down
WRAP53	-5.49	48.72	0.0000982	0.0144	Down
MUC5AC	-5.45	687.67	0.0000971	0.0144	Down
FAN1	-5.45	68.34	0.0014	0.0144	Down
PLA2G6	-5.43	44.07	0.0000675	0.0146	Down
ZSCAN25	-5.42	138.16	0.0000016	0.0146	Down
ARMC5	-5.40	95.43	0.00000841	0.0147	Down
PATZ1	-5.39	53.77	0.00133	0.0148	Down
OAS3	-5.38	192.46	3.42E-08	0.0148	Down
NAA40	-5.37	17.68	0.000365	0.015	Down
SKI	-5.36	25.00	0.00033	0.015	Down
NOD1	-5.36	35.88	0.000422	0.015	Down
AP4B1	-5.34	20.91	0.00115	0.0156	Down
DHRS11	-5.34	70.50	0.00309	0.0156	Down
PHRF1	-5.31	90.82	6.62E-08	0.016	Down
COPG1	-5.31	98.22	0.00000287	0.016	Down
HECTD3	-5.31	43.70	0.000636	0.016	Down
ARHGEF17	-5.30	131.40	1.29E-08	0.016	Down
SIPA1L3	-5.30	133.42	0.000031	0.0161	Down
TNK1	-5.30	47.26	0.000225	0.0161	Down
ZC3H3	-5.30	54.02	0.00112	0.0167	Down
TEX22	-5.30	27.36	0.0018	0.0169	Down
AP5B1	-5.29	78.72	0.000108	0.017	Down
POLG	-5.28	118.98	0.000151	0.017	Down
NCKIPSD	-5.26	37.71	0.0000391	0.0172	Down
NLRC3	-5.26	78.87	0.000881	0.0173	Down
PCDHGA7	-5.26	74.89	0.00126	0.0173	Down
WNK2	-5.25	217.97	0.00000867	0.0174	Down
APBB1	-5.25	40.78	0.00334	0.0177	Down
SLC29A4	-5.21	15.30	0.00237	0.0177	Down

PRR12	-5.20	39.74	0.00194	0.0185	Down
CCDC18	-5.18	29.20	0.00106	0.0186	Down
STUM	-5.18	96.30	0.00271	0.0186	Down
FANCA	-5.17	139.59	0.000000712	0.0189	Down
FAM168A	-5.16	25.38	0.000809	0.0189	Down
LRRC20	-5.16	44.35	0.00103	0.019	Down
PCDHGA2	-5.16	70.29	0.00143	0.0191	Down
COL1A1	-5.13	58.34	0.000032	0.0192	Down
GRIN1	-5.13	76.17	0.00114	0.0192	Down
ADARB1	-5.13	30.41	0.00207	0.0193	Down
CMTR1	-5.11	98.08	0.000000521	0.0193	Down
PEX6	-5.11	61.89	0.000938	0.0195	Down
PCDHGB1	-5.11	67.74	0.00154	0.0195	Down
PCDHGB3	-5.11	67.74	0.00154	0.0195	Down
BEGAIN	-5.11	22.39	0.00303	0.0196	Down
SLC49A3	-5.10	26.96	0.00043	0.0196	Down
BUD13	-5.10	10.94	0.0011	0.0197	Down
PCDHGC4	-5.10	67.08	0.00147	0.0197	Down
ITGAM	-5.09	100.97	0.00000743	0.0197	Down
TTN	-5.09	334.49	0.000194	0.02	Down
PCDHGA4	-5.09	66.71	0.00159	0.0203	Down
HDAC9	-5.09	23.10	0.002	0.0205	Down
PCDHGA12	-5.08	66.20	0.00162	0.0205	Down
TTL	-5.07	44.24	0.000216	0.0206	Down
PHLPP1	-5.06	24.82	0.00019	0.0206	Down
UBE2C	-5.05	33.76	0.000998	0.021	Down
PCDHGA9	-5.05	65.18	0.00167	0.021	Down
ARHGAP39	-5.04	51.03	0.0000425	0.0212	Down
ECM1	-5.04	44.38	0.0000608	0.0212	Down
CC2D1A	-5.04	44.73	0.000127	0.0212	Down
PCDHGC3	-5.04	64.52	0.000272	0.0213	Down
SPEG	-5.03	102.42	0.00173	0.0213	Down
CPAMD8	-5.02	38.46	0.0000199	0.0215	Down
H6PD	-5.02	113.98	0.000228	0.0216	Down
TRIM36	-5.01	24.25	0.00131	0.0216	Down
FKBP9	-4.97	61.01	0.000000539	0.0219	Down
LMTK2	-4.95	136.22	1.46E-08	0.0219	Down
CLPB	-4.95	64.75	0.000961	0.0222	Down
NIBAN2	-4.94	91.39	4.02E-09	0.0223	Down
PCDHGB2	-4.93	60.07	0.00197	0.0224	Down
PCDHGA5	-4.93	60.07	0.00197	0.0224	Down
CDIP1	-4.92	44.40	0.000487	0.0225	Down
PCDHGA8	-4.90	58.60	0.00204	0.0225	Down
FBXL19	-4.90	20.06	0.00243	0.0227	Down
CHAD	-4.89	42.15	0.00109	0.0229	Down
PCDHGC5	-4.89	58.38	0.00194	0.0231	Down
ZNF618	-4.87	89.59	0.000167	0.0233	Down
ZNF710	-4.87	46.23	0.000242	0.0233	Down
AMH	-4.87	42.77	0.000658	0.0235	Down
ENSG0000028[illegible]	-4.84	41.69	0.000028	0.0236	Down

MCF2L	-4.84	129.57	0.000155	0.0239	Down
SLC28A3	-4.84	22.29	0.00173	0.024	Down
PCDHGA3	-4.84	56.50	0.00224	0.0242	Down
IDUA	-4.83	37.42	0.00036	0.0242	Down
SRRM3	-4.83	38.59	0.00314	0.0242	Down
MTO1	-4.82	39.19	0.000492	0.0242	Down
STS	-4.82	39.68	0.000835	0.0244	Down
CGAS	-4.82	40.64	0.00151	0.025	Down
PCDHGA1	-4.82	55.48	0.00233	0.0255	Down
PCDHGA6	-4.82	55.48	0.00233	0.0255	Down
PCDHGB7	-4.82	55.48	0.00233	0.0255	Down
MUC16	-4.81	371.23	0.0000413	0.0256	Down
BICRA	-4.81	24.66	0.000918	0.0256	Down
UBN2	-4.80	51.67	0.00017	0.0258	Down
NUP205	-4.79	72.88	0.000143	0.0261	Down
SRPX2	-4.79	46.81	0.000396	0.0261	Down
RPUSD3	-4.78	40.44	0.00174	0.0261	Down
ZWINT	-4.77	36.98	0.00279	0.0261	Down
CENPT	-4.76	52.02	0.00279	0.0261	Down
PARP10	-4.73	36.31	0.0000525	0.0261	Down
TNKS1BP1	-4.71	66.09	0.0000074	0.0264	Down
FOXC1	-4.70	16.22	0.000541	0.0268	Down
WDR81	-4.69	38.49	0.00016	0.0269	Down
ENGASE	-4.67	42.42	0.000809	0.0269	Down
CDKAL1	-4.66	28.51	0.000317	0.0269	Down
SLC9A1	-4.66	70.51	0.000591	0.0269	Down
LIMD1	-4.63	91.39	0.000000115	0.0271	Down
KAT2B	-4.62	9.93	0.00336	0.0274	Down
RAPGEF1	-4.61	80.44	0.000057	0.0274	Down
ZFYVE27	-4.60	53.95	0.000117	0.0279	Down
ZNF549	-4.59	75.21	0.00245	0.028	Down
KIF1A	-4.59	72.48	0.00333	0.028	Down
ATP7B	-4.57	132.76	0.0000381	0.028	Down
IBA57	-4.57	57.69	0.00286	0.0281	Down
ADGRA2	-4.56	42.44	0.00102	0.0282	Down
CCDC9	-4.55	47.06	0.00044	0.0283	Down
FAM126B	-4.54	28.02	0.000109	0.0283	Down
SRCIN1	-4.54	35.14	0.0032	0.0287	Down
PACS1	-4.52	39.42	0.000335	0.0288	Down
BCL3	-4.50	65.64	0.0000283	0.029	Down
CAPN15	-4.50	160.43	0.0000832	0.0297	Down
ZBTB7B	-4.48	60.22	0.000315	0.0297	Down
CARS2	-4.48	129.78	0.003	0.0301	Down
MAP3K14	-4.44	68.46	0.000419	0.0301	Down
VARS2	-4.43	44.45	0.000651	0.0301	Down
EXT1	-4.42	71.23	0.0000598	0.0305	Down
CDK5RAP2	-4.42	113.67	0.000176	0.0308	Down
E2F4	-4.42	26.75	0.0018	0.0308	Down
STON2	-4.36	104.04	0.000108	0.0308	Down
PLEKHG2	-4.35	78.98	0.0000248	0.0308	Down

ZNF8	-4.35	31.74	0.00259	0.031	Down
CARD10	-4.34	48.46	0.000606	0.0311	Down
TNFRSF21	-4.33	67.43	0.000264	0.0311	Down
CXCR5	-4.30	44.14	0.000505	0.0311	Down
FBRSL1	-4.29	80.98	0.00043	0.0317	Down
ASPSCR1	-4.29	73.31	0.000908	0.0317	Down
CNTRL	-4.29	35.87	0.00279	0.0317	Down
RIOX1	-4.28	24.78	0.0021	0.0321	Down
C19orf44	-4.28	16.11	0.00334	0.0322	Down
SPTLC3	-4.26	101.02	0.0007	0.0322	Down
SOGA1	-4.26	109.38	0.00122	0.0322	Down
SGSH	-4.25	191.26	0.0000405	0.0325	Down
PSKH1	-4.24	83.80	0.00022	0.0325	Down
VPS53	-4.24	127.09	0.000358	0.0325	Down
DHX37	-4.23	141.37	0.00218	0.0325	Down
ATAD2	-4.20	182.02	0.000434	0.0326	Down
PNPLA7	-4.19	63.06	0.00127	0.0327	Down
POLR1A	-4.17	112.75	0.0000233	0.0329	Down
EPHB4	-4.17	100.17	0.00306	0.0332	Down
ITGA5	-4.16	49.41	0.0028	0.0332	Down
TCOF1	-4.14	189.01	0.000315	0.0332	Down
AKT2	-4.14	113.56	0.00122	0.0332	Down
EPN1	-4.13	148.33	0.000227	0.034	Down
RELB	-4.12	161.04	0.0000276	0.0345	Down
WDR75	-4.10	71.22	0.00267	0.0347	Down
MLH1	-4.08	126.69	0.00025	0.0349	Down
CMIP	-4.07	125.17	0.00104	0.0353	Down
FBXL8	-4.07	45.78	0.0017	0.0354	Down
SART1	-4.06	56.95	0.00175	0.0354	Down
ZNF544	-4.04	60.20	0.00209	0.0357	Down
TNK2	-4.03	65.49	0.000205	0.0357	Down
RAB11FIP3	-4.03	90.63	0.00205	0.0357	Down
TTLL3	-4.02	163.10	0.000847	0.036	Down
TNS2	-4.02	64.84	0.00306	0.0361	Down
ITPKB	-4.02	34.65	0.00331	0.0363	Down
INF2	-4.01	265.43	0.00000218	0.0364	Down
KLHL25	-4.00	67.41	0.000571	0.0366	Down
WNT7B	-3.99	156.37	0.00294	0.0367	Down
FBXO42	-3.98	34.07	0.00294	0.0368	Down
DAB2IP	-3.96	68.13	0.000869	0.0368	Down
RAB11B	-3.95	75.48	0.000015	0.0368	Down
TRIM26	-3.94	161.05	0.0000617	0.0369	Down
VPS26C	-3.94	142.72	0.000966	0.0369	Down
SMURF2	-3.94	35.63	0.00113	0.0369	Down
ENSG00000284	-3.94	34.75	0.00202	0.0369	Down
ANKRD27	-3.90	51.51	0.00157	0.0369	Down
PPP1R13L	-3.89	61.78	0.000235	0.0371	Down
IQANK1	-3.88	64.07	0.00133	0.0371	Down
CABIN1	-3.83	122.73	0.00322	0.0375	Down
DENND3	-3.81	402.94	0.000181	0.0376	Down

PHF2	-3.81	82.58	0.000217	0.0377	Down
SEMA4C	-3.79	173.44	0.000367	0.0378	Down
GLIS2	-3.78	64.60	0.000268	0.038	Down
TYMS	-3.78	67.40	0.00216	0.038	Down
KDM6B	-3.77	222.48	0.0000331	0.038	Down
PTPN23	-3.75	152.81	0.000156	0.038	Down
BRAF	-3.75	54.96	0.00023	0.0382	Down
PAQR4	-3.75	245.23	0.000584	0.0382	Down
PRDM10	-3.75	27.01	0.00332	0.0384	Down
SERINC2	-3.73	165.02	0.000452	0.0384	Down
TBCD	-3.70	262.73	0.000475	0.0385	Down
MGAT1	-3.70	124.57	0.000531	0.0385	Down
LARGE1	-3.69	197.32	0.000263	0.0385	Down
ATG2A	-3.69	101.92	0.000382	0.0385	Down
ERN1	-3.69	119.53	0.000457	0.0389	Down
ZNF839	-3.69	24.97	0.00134	0.0389	Down
TPX2	-3.68	40.16	0.000955	0.0394	Down
DUS3L	-3.68	35.22	0.00188	0.0394	Down
PTPRU	-3.67	92.18	0.000338	0.0394	Down
ACAD9	-3.66	131.20	0.000453	0.0394	Down
MBD1	-3.63	72.30	0.00199	0.0396	Down
SOCS3	-3.63	47.20	0.00239	0.0396	Down
CLN6	-3.62	66.53	0.000233	0.0396	Down
STRN4	-3.60	62.41	0.00163	0.0397	Down
REEP1	-3.60	22.71	0.00239	0.0398	Down
TYK2	-3.58	99.72	0.000821	0.0398	Down
MAN2A2	-3.57	156.81	0.0000993	0.0398	Down
WEE1	-3.57	127.71	0.00127	0.0399	Down
FOXK1	-3.56	86.56	0.000167	0.0399	Down
CHERP	-3.56	37.33	0.0014	0.0405	Down
BAP1	-3.55	64.24	0.00228	0.0407	Down
WDR24	-3.54	53.41	0.00269	0.0407	Down
ACVR1B	-3.50	82.10	0.000983	0.0407	Down
ANKFY1	-3.49	155.47	0.000749	0.0407	Down
CBL	-3.48	206.54	0.0000104	0.0408	Down
ZNF629	-3.48	69.00	0.000239	0.0408	Down
ANAPC7	-3.48	45.88	0.00167	0.0413	Down
NOC4L	-3.45	26.06	0.00228	0.0413	Down
PRDM2	-3.45	133.16	0.00234	0.0422	Down
BAHD1	-3.45	135.54	0.00334	0.0422	Down
MAN2C1	-3.44	98.32	0.000545	0.0424	Down
ITPR3	-3.43	211.05	0.000244	0.0424	Down
CDC42BPB	-3.42	370.32	0.000511	0.0431	Down
LETM1	-3.39	110.83	0.00111	0.0435	Down
USP36	-3.37	122.02	0.000102	0.0437	Down
TMED8	-3.36	32.05	0.00222	0.0438	Down
WDFY3	-3.34	136.76	0.000825	0.0438	Down
SIDT2	-3.34	90.79	0.00115	0.0438	Down
RTEL1-TNFRSF6	-3.34	113.48	0.00146	0.0444	Down
NOL4L	-3.33	196.98	0.000357	0.0444	Down

LGALS3BP	-3.32	396.62	0.000916	0.0446	Down
XRCC3	-3.30	140.32	0.00247	0.0446	Down
MLXIP	-3.29	220.94	0.00176	0.0446	Down
BTBD1	-3.27	60.88	0.00136	0.0446	Down
FAM222A	-3.27	60.92	0.0033	0.0446	Down
FLYWCH1	-3.26	75.98	0.00171	0.0451	Down
TBC1D9B	-3.25	259.64	0.000446	0.0451	Down
ENSG0000024[illegible]	-3.24	61.89	0.00275	0.0451	Down
GBP3	-3.23	61.72	0.00318	0.0451	Down
C2CD2L	-3.21	62.04	0.00257	0.0451	Down
NMT1	-3.20	123.86	0.000557	0.0451	Down
CCAR2	-3.20	239.09	0.000581	0.0451	Down
TMEM132A	-3.20	117.21	0.00136	0.0451	Down
RAB11FIP4	-3.19	208.59	0.00235	0.0452	Down
FOXP4	-3.18	97.22	0.00141	0.0457	Down
PI4KA	-3.17	198.30	0.0000457	0.046	Down
DDX41	-3.10	95.60	0.000121	0.046	Down
CIC	-3.07	149.88	0.00237	0.046	Down
ZNF335	-3.00	200.80	0.00185	0.046	Down
IPO4	-3.00	132.39	0.0021	0.0461	Down
GBF1	-2.99	178.82	0.000213	0.0464	Down
ATP13A2	-2.98	65.56	0.00287	0.0464	Down
ENOSF1	-2.97	260.75	0.00202	0.0466	Down
TP53	-2.97	101.23	0.00271	0.0466	Down
RANBP10	-2.97	58.32	0.00336	0.0466	Down
CLUH	-2.93	145.60	0.000486	0.0468	Down
MAPK8IP3	-2.92	326.77	0.000135	0.0471	Down
MED15	-2.90	137.05	0.000833	0.0472	Down
RIF1	-2.89	124.85	0.00206	0.0474	Down
ESRP2	-2.88	86.46	0.00174	0.0474	Down
TESK1	-2.88	38.44	0.00223	0.0476	Down
NUP98	-2.86	173.01	0.00129	0.0481	Down
CNN2	-2.83	186.48	0.000313	0.0482	Down
WDR90	-2.82	437.06	0.00196	0.0485	Down
SREBF2	-2.82	170.62	0.00224	0.0486	Down
USP5	-2.81	69.85	0.0025	0.0487	Down
NUP133	-2.76	120.77	0.00294	0.0487	Down
SETD1B	-2.75	43.78	0.00285	0.0489	Down
SCNN1A	-2.74	455.24	0.00208	0.0495	Down
CRAMP1	-2.71	147.92	0.00284	0.0499	Down
SMAD3	-2.69	231.65	0.000665	0.0499	Down
PKMYT1	-2.68	278.98	0.00214	0.0499	Down
SLC7A1	-2.68	96.28	0.00301	0.0499	Down

Supplementary Table 2: Genes upregulated in CTCs post-digoxin.

Table listing the genes upregulated in CTC pools obtained 32 days post-digoxin intake (n = 2) compared to CTC pools obtained at 86 days, 17 days and zero days prior to digoxin intake (n = 5) in patient 5. Gene ID, fold change, P value, and adjusted P value are shown (edgeR likelihood ratio test for differential expression, with Benjamini-Hochberg adjustment).

**Gene ID**	**Log2FoldChang**	**Base Mean**	***P* value**	**Adjusted *p* val**	**Direction**
TRMT112	3.42	103.77	0.0000941	0.00417	Up
MRPS31	7.37	14.98	0.000196	0.00691	Up
NAT1	3.22	118.87	0.000258	0.00811	Up
AARD	5.30	85.56	0.000484	0.0128	Up
NIPSNAP3A	4.91	20.70	0.000496	0.013	Up
GSTM3	3.46	58.61	0.000608	0.0151	Up
SCGB1D2	4.72	28.13	0.000613	0.0151	Up
PTS	3.18	60.99	0.000677	0.0164	Up
CA2	4.17	63.06	0.00139	0.0279	Up
ATP6V1G1	2.60	212.88	0.00142	0.0282	Up
GDPD3	3.81	25.67	0.0015	0.0294	Up
ENSG0000028	2.70	148.47	0.0016	0.0308	Up
CIAO2A	2.68	88.04	0.00171	0.0322	Up
MED4	3.44	50.87	0.00184	0.0339	Up
LAMTOR5	2.89	178.07	0.00225	0.0385	Up
ATP5IF1	2.53	85.49	0.00228	0.0389	Up
MIA-RAB4B	4.14	142.66	0.00229	0.0389	Up
BID	3.38	73.66	0.00263	0.0429	Up
HSBP1	2.34	223.35	0.00263	0.0429	Up
IFT20	3.87	22.08	0.00277	0.0445	Up
SELENOK	3.26	74.19	0.00296	0.0462	Up
PDIA3	2.15	489.60	0.00305	0.0471	Up
NDUFB3	3.16	67.27	0.00325	0.0493	Up

Supplementary Data 1.

Effect of Digoxin on clusters of circulating tumor cells (CTCs) in breast cancer patients

Clinical Study Protocol

Prospective therapeutic exploratory trial on the effect of digoxin on clusters of circulating tumor cells in patients with advanced or metastasized breast cancer

DICCT

Study Type:	Therapeutic exploratory clinical trial with Investigational Medicinal Product (IMP)
Study Categorisation:	Risk category according to HRA B
Study Registration:	U.S. National Institutes of Health clinical trial registry (clinicaltrials.gov)
Sponsor:	University Hospital Basel / Prof Dr Christian Kurzeder<br>Spitalstrasse 21<br>4031 Basel
Coordinating Investigator	PD Dr Marcus Vetter, Mühlemattstrasse 26, 4410 Liestal
Principal Investigator(s)	USB: Prof Dr Christian Kurzeder, Spitalstrasse 2, 4031 Basel (Sponsor-Investigator)<br>KSBL: Dr med. Angela Kohler, Mühlemattstrasse 26, 4410 Liestal<br>USZ: Dr med. Bich Doan Nguyen-Sträuli, Frauenklinikstrasse 10, 8091 Zürich
Investigational Product:	Digoxin
Protocol Version and Date:	2.4_23.01.2023

CONFIDENTIAL

e.g. “The information contained in this document is confidential and the property of the sponsor. The information may not - in full or in part - be transmitted, reproduced, published, or disclosed to others than the applicable Competent Ethics Committee(s) and Regulatory Authority(ies) without prior written authorisation from the sponsor except to the extent necessary to obtain informed consent from those who will participate in the study.

Signature Page(s)

Study number	U.S. National Institutes of Health clinical trial registry (clinicaltrials.gov)
Study Title	Effect of Digoxin on clusters of circulating tumor cells (CTCs) in breast cancer patients

The Sponsor-Investigator and trial statistician have approved the protocol version 2.4 (dated 23.01.2023), and confirm hereby to conduct the study according to the protocol, current version of the World Medical Association Declaration of Helsinki, ICH-GCP guidelines or ISO 14155 norm if applicable and the local legally applicable requirements.

Sponsor:
Prof Dr Christian Kurzeder, Spitalstrasse 21, 4031 Basel

23.1.23

Place/Date Signature

Trial Statistician:
Sabine Schädelin
Department of Clinical Research
Spitalstrasse 12
4031 Basel
Switzerland

27.1.23

Place/Date Signature

Local Principal Investigator at study site*:

I have read and understood this trial protocol and agree to conduct the trial as set out in this study protocol, the current version of the World Medical Association Declaration of Helsinki, ICH-GCP guidelines or ISO 14155 norm and the local legally applicable requirements.

Site	Breast Cancer Center, University Hospital Basel, Spitalstrasse 21, 4031 Basel, Switzerland
Principal investigator/ Sponsor Investigator	Prof Dr Christian Kurzeder; Spitalstrasse 21, 4031 Basel

23.1.23

Place/Date

Signature

Note: In multicentre studies, this page must be individually signed by all participating Local Principal

Effect of Digoxin on clusters of circulating tumor cells (CTCs) in breast cancer patients

Clinical Study Protocol

Prospective therapeutic exploratory trial on the effect of digoxin on clusters of circulating tumor cells in patients with advanced or metastasized breast cancer

DICCT

Study Type:	Therapeutic exploratory clinical trial with Investigational Medicinal Product (IMP)
Study Categorisation:	Risk category according to HRA B
Study Registration:	U.S. National Institutes of Health clinical trial registry (clinicaltrials.gov)
Sponsor:	University Hospital Basel / Prof Dr Christian Kurzeder<br>Spitalstrasse 21<br>4031 Basel
Coordinating Investigator	PD Dr Marcus Vetter, Mühlemattstrasse 26, 4410 Liestal
Principal Investigator(s)	USB: Prof Dr Christian Kurzeder, Spitalstrasse 2, 4031 Basel (Sponsor-Investigator)<br>KSBL: Dr med. Angela Kohler, Mühlemattstrasse 26, 4410 Liestal<br>USZ: Dr med. Bich Doan Nguyen-Sträuli, Frauenklinikstrasse 10, 8091 Zürich
Investigational Product:	Digoxin
Protocol Version and Date:	2.4_23.01.2023

CONFIDENTIAL

e.g. "The information contained in this document is confidential and the property of the sponsor. The information may not - in full or in part - be transmitted, reproduced, published, or disclosed to others than the applicable Competent Ethics Committee(s) and Regulatory Authority(ies) without prior written authorisation from the sponsor except to the extent necessary to obtain informed consent from those who will participate in the study.

Local Principal Investigator at study site*:

I have read and understood this trial protocol and agree to conduct the trial as set out in this study protocol, the current version of the World Medical Association Declaration of Helsinki, ICH-GCP guidelines or ISO 14155 norm and the local legally applicable requirements.

Site	Oncology, Haematology & Immuntherapy, Kantonsspital Baselland, Liestal, Mühlemattstrasse 26, 4410 Liestal, Switzerland
Principal investigator	Dr med. Angela Kohler, Mühlemattstrasse 26, 4410 Liestal

Liestal, 24.01.23

Place/Date Signature

Note: In multicentre studies, this page must be individually signed by all participating Local Principal Investigators.

Local Principal Investigator at study site*:

I have read and understood this trial protocol and agree to conduct the trial as set out in this study protocol, the current version of the World Medical Association Declaration of Helsinki, ICH-GCP guidelines or ISO 14155 norm and the local legally applicable requirements.

Site	Breast Cancer Unit, Clinic for Gynecology, University Hospital Zurich, Frauenklinikstrasse 10, 8091 Zurich, Switzerland
Principal investigator	Dr med. Bich Doan Nguyen-Sträuli, Frauenklinikstrasse 10, 8091 Zurich

Zürich, 24.01.23

Place/Date

Signature

Note: In multicentre studies, this page must be individually signed by all participating Local Principal Investigators.

Table of Contents

STUDY SYNOPSIS **9**
ABBREVIATIONS **16**
STUDY SCHEDULE **17**
1. STUDY ADMINISTRATIVE STRUCTURE **18**
1.1 Sponsor, Sponsor-Investigator 18
1.2 Principal Investigator(s) 18
1.3 Statistician ("Biostatistician") 18
1.4 Laboratory 18
1.5 Monitoring institution 18
1.6 Data Safety Monitoring Committee 19
1.7 Any other relevant Committee, Person, Organisation, Institution 19
2. ETHICAL AND REGULATORY ASPECTS **19**
2.1 Study registration 19
2.2 Categorisation of study 19
2.3 Competent Ethics Committee (CEC) 19
2.4 Competent Authorities (CA) 19
2.5 Ethical Conduct of the Study 20
2.6 Declaration of interest 20
2.7 Patient Information and Informed Consent 20
2.8 Participant privacy and confidentiality 20
2.9 Early termination of the study 20
2.10 Protocol amendments 21
3. BACKGROUND AND RATIONALE **21**
3.1 Background and Rationale 21
3.2 Investigational Product (treatment, device) and Indication 21
3.3 Preclinical Evidence 21
3.4 Clinical Evidence to Date 22
3.5 Dose Rationale 24
3.6 Explanation for choice of comparator (or placebo) 24
3.7 Risks / Benefits 24
3.8 Justification of choice of study population 24
4. STUDY OBJECTIVES **25**
4.1 Overall Objective 25
4.2 Primary Objective 25
4.3 Secondary Objectives 25
4.4 Safety Objectives 25
5. STUDY OUTCOMES **25**
5.1 Primary Outcome 25
5.2 Secondary Outcomes 25
5.3 Other Outcomes of Interest 25
5.4 Safety Outcomes 25
6. STUDY DESIGN **26**
6.1 General study design and justification of design 26
6.2 Methods of minimising bias 26
6.2.1 Randomisation 26

6.2.2 Blinding procedures 26
6.2.3 Other methods of minimising bias 26
6.3 Unblinding Procedures (Code break) 26
7. STUDY POPULATION 26
7.1 Eligibility criteria 26
7.2 Recruitment and screening 27
7.3 Assignment to study groups 27
7.4 Criteria for withdrawal / discontinuation of participants 27
8. STUDY INTERVENTION 28
8.1 Identity of Investigational Products (treatment / medical device) 28
8.1.1 Experimental Intervention (treatment / medical device) 28
8.1.2 Control Intervention (standard/routine/comparator treatment / medical device) 28
8.1.3 Packaging, Labelling and Supply (re-supply) 28
8.1.4 Storage Conditions 28
8.2 Administration of experimental and control interventions 28
8.2.1 Experimental Intervention 28
8.2.2 Control Intervention 28
8.3 Dose / Device modifications 28
8.4 Compliance with study intervention 28
8.5 Data Collection and Follow-up for withdrawn participants 28
8.6 Trial specific preventive measures 29
8.7 Concomitant Interventions (treatments) 29
8.8 Study Drug / Medical Device Accountability 29
8.9 Return or Destruction of Study Drug / Medical Device 29
9. STUDY ASSESSMENTS 30
9.1 Study flow chart(s) / table of study procedures and assessments 30
9.2 Assessments of outcomes 30
9.2.1 Assessment of primary outcome 30
9.2.2 Assessment of secondary outcomes 30
9.2.3 Assessment of other outcomes of interest 30
9.2.4 Assessment of safety outcomes 30
9.2.5 Assessments in participants who prematurely stop the study 30
9.3 Procedures at each visit 31
9.3.1 Screening visit 31
9.3.2 Visit 2, 3, 5, 7 31
9.3.3 Visit 4, 6, 8 31
10. SAFETY 32
10.1 Drug studies 32
10.1.1 Definition and assessment of (serious) adverse events and other safety related events 32
10.1.2 Reporting of serious adverse events (SAE) and other safety related events 33
10.1.3 Follow up of (Serious) Adverse Events 33
10.2 Medical Device Category C studies 34
10.3 Medical Device Category A studies 34
10.4 Assessment, notification and reporting on the use of radiation sources 34
11. STATISTICAL METHODS 35
11.1 Hypothesis 35

11.2 Determination of Sample Size 35
11.3 Statistical criteria of termination of trial 36
11.4 Planned Analyses 36
11.4.1 Datasets to be analysed, analysis populations 36
11.4.2 Demographic and baseline characteristics 36
11.4.3 Primary Analysis 36
11.4.4 Secondary Analyses 36
11.4.5 Interim analyses 36
11.4.6 Safety analysis 36
11.4.7 Deviation(s) from the original statistical plan 36
11.5 Handling of missing data and drop-outs 36
12. QUALITY ASSURANCE AND CONTROL 38
12.1 Data handling and record keeping / archiving 38
12.1.1 Case Report Forms 38
12.1.2 Specification of source documents 38
12.1.3 Record keeping / archiving 38
12.2 Data management 38
12.2.1 Data Management System 38
12.2.2 Data security, access and back-up 39
12.2.3 Analysis and archiving 39
12.2.4 Electronic and central data validation 39
12.3 Monitoring 39
12.4 Audits and Inspections 39
12.5 Confidentiality, Data Protection 39
12.6 Storage of biological material and related health data 40
13. PUBLICATION AND DISSEMINATION POLICY 40
14. FUNDING AND SUPPORT 40
14.1 Funding 40
14.2 Other Support 40
15. INSURANCE 40
16. REFERENCES 41
17. APPENDICES 43

STUDY SYNOPSIS

**Sponsor**	University Hospital Basel/, Prof Dr Christian Kurzeder
**Principal investigator(s)**	USB: Prof Dr Christian Kurzeder ; Spitalstrasse 21, 4031 Basel<br>KSBL: Dr med. Angela Kohler, Mühlemattstrasse 26, 4410 Liestal<br>USZ: Dr med. Bich Doan Nguyen-Sträuli, Frauenklinikstrasse 10, 8091 Zürich
**Study Title:**	Effect of Digoxin on clusters of circulating tumor cells in breast cancer patients
**Short Title / Study ID:**	Digoxin induced dissolution of CTC clusters
**Protocol Version and Date:**	Version 2.4<br>23.01.2023
**Trial registration:**	Planned at U.S. National Institutes of Health clinical trial registry (clinicaltrials.gov)
**Study category and Rationale**	Category B<br>Digoxin is authorized in Switzerland for treatment of heart failure and supraventricular tachycardia. Dosage and target serum level of digoxin in this trial are in accordance with the label
**Clinical Phase:**	Therapeutic exploratory
**Background and Rationale:**	CTCs are considered to be precursors of metastasis in various cancer types and are found in the blood of cancer patients as single CTCs and CTC clusters, with the latter featuring a higher ability to seed metastasis. CTC cluster share several properties that commonly feature stem cell biology which drive metastases formation.<br>Preclinical data shows that CTC clusters can be disaggregated into single cells by the treatment with Na+/K+ ATPase inhibitors such as digoxin. In xenograft models disruption of CTC clusters into single cells dramatically decreases spontaneous metastasis formation (80-fold). Treatment of cancer patients with Digoxin could therefore be a valuable therapeutic strategy.

**Objective(s):**	Primary objective:<br>• To assess the effect of digoxin on the size of CTC clusters in patients with advanced breast cancer<br>Secondary objectives:<br>• To assess the effect of digoxin on the number of CTC clusters detected in patients with advanced breast cancer<br>• To investigate the kinetics of dissolution of CTC clusters<br>• To investigate the dose response relationship of the effect<br>Exploratory objective:<br>• To Investigate mean CTC cluster size after initiation of anticancer therapy
**Outcome(s):**	Primary study outcome:<br>• mean CTC cluster size<br>Secondary study outcomes:<br>• mean CTC cluster number<br>• Mean time to dissolution of CTC clusters
**Study design:**	This will be a single arm therapeutic exploratory study of digoxin in patients with advanced or metastatic breast cancer.<br>Patients with advanced or metastatic breast cancer in whom CTC clusters could be identified will receive an individualized daily maintenance dose of digoxin adapted to their kidney function. Blood samples for analyses of digoxin serum level and mean CTC cluster size will be drawn at specified time points.

**Inclusion / Exclusion criteria:**	Inclusion criteria:<br>• Informed Consent as documented by signature (Appendix Informed Consent Form)<br>• Adult women and men (≥ 18 years of age) with proven diagnosis of adenocarcinoma of the breast with evidence of locoregionally recurrent or metastatic disease not amenable to resection, radiation therapy or systemic therapy with curative intent<br>• Adequate organ and marrow function defined as follows:<br>• ANC ≥1,500/mm3 (1.5 x 109 /L);<br>• Platelets ≥100,000/mm3 (100 x 109 /L);<br>• Hemoglobin ≥9 g/dL (90 g/L);<br>• Serum creatinine ≤1.5 x ULN or estimated creatinine clearance ≥ 60 mL/min as calculated using the method standard for the institution;<br>• Total serum bilirubin ≤1.5 x ULN (≤3.0 x ULN if Gilbert’s disease);<br>• AST and/or ALT ≤3 x ULN (≤5.0 x ULN if liver metastases present);<br>• Alkaline phosphatase ≤2.5 x ULN (≤5.0 x ULN if bone or liver metastases present).<br>• Resolution of all acute toxic effects of prior anti-cancer therapy or surgical procedures to NCI CTCAE version 4.0 Grade ≤1 (except alopecia or other toxicities not considered a safety risk for the patient at investigator's discretion).<br>Exclusion:<br>• Patients on treatment with digoxin or digitoxin<br>• Patients with atrial fibrillation or atrial flutter<br>• Ventricular Fibrillation or ventricular tachycardia,<br>• Atrioventricular heart block 2nd or 3rd degree, sick sinus syndrome or sinus bradycardia,<br>• Wolff-Parkinson-White Syndromeor<br>• Hypokalemia, hypercalcemia, hypomagnesemia,<br>• Hypoxia,<br>• hypertrophic cardiomyopathy,<br>• aortic aneurysm,<br>• simultaneous intravenous application of calcium salts<br>• Known hypersensitivity to Digoxin, other cardiac glycosides or included compounds

	• Known drug interactions of ongoing cancer therapy with digoxin<br>• Women who are pregnant or breast feeding,<br>• Intention to become pregnant during the course of the study,
**Inclusion / Exclusion criteria:**	• Lack of safe contraception, defined as: Female participants of childbearing potential, not using and not willing to continue using a medically reliable method of contraception for the entire study duration, such as oral, injectable, or implantable contraceptives, or intrauterine contraceptive devices, or who are not using any other method considered sufficiently reliable by the investigator in individual cases.<br>• Inability to follow the procedures of the study, e.g. due to language problems, psychological disorders, dementia, etc. of the participant<br>• Participation in another study with investigational drug within the 30 days preceding and during the present study,<br>• Female participants who are surgically sterilised / hysterectomised or post-menopausal for longer than 2 years are not considered as being of child bearing potential.<br>• Other clinically significant concomitant disease states (e.g., renal failure, hepatic dysfunction, cardiovascular disease, etc.),<br>• Known or suspected non-compliance, drug or alcohol abuse<br><br>Exclusion:<br>• Previous enrolment into the current study,<br>• Enrolment of the investigator, his/her family members, employees and other dependent persons,
**Measurements and procedures:**	Only patients in whom at least one cluster of CTCs is detectable at screening will enter treatment phase.<br>Blood samples for analyses of mean CTC cluster size will be drawn at screening, on day 0 (prior to digoxin intake (control) and 2 hrs after first oral intake), on day 3 and on day 7. Patients will enter up to two more weekly cycles with two more blood samples taken within each cycle
**Study Product / Intervention:**	Patients will receive a daily maintenance dose of digoxin. The daily dose of digoxin will be calculated according to the renal function and the target serum digoxin concentration and applied in an adjusted regimen based on the availability of 0.125 mg and 0.25 mg pills in the morning (before 10 am). Blood samples for analyses of mean CTC cluster size will be drawn at screening, on day 0 (2 hrs after first oral intake), on day 3 and on day 7. Depending on the digoxin serum level maintenance therapy with digoxin will be continued up to 3 weeks if the digoxin serum level on day 7 or day 14 is below 0.70 ng/ml. For the third week of maintenance therapy individual dose adjustments will be carried out as needed.
**Control Intervention (if applicable):**	Blood samples drawn before IMP administration for analyses of mean CTC cluster size will serve as a control

**Number of Participants with Rationale:**	Appr. 50 – 60 patients will be screened for CTC clusters<br><br>Expected number of patients with detectable CTC clusters: n=~12 (25%)<br><br>Expected number of patients with a Digoxin serum level within target range after up to 3 weeks of trial therapy: n=9 (80%). Accrual of patients until in n=9 patients a Digoxin serum level within target range has been reached.<br><br>The calculated sample size of 9 allows with a power of 0.8 to estimate a mean treatment effect of digoxin of 1.1 (average CTC-cluster size reduction in cell equivalents).
**Study Duration:**	Estimated accrual duration: 24 months<br><br>Duration of trial therapy (per patient): up to 3 weeks<br><br>Follow up: no systematic follow up, 1 visit after start of next line of systemic therapy<br><br>Estimated trial duration in total: 24 months
**Study Schedule:**	05/19 First-Participant-In (planned)<br><br>05/23 Last-Participant-Out (planned)

**Investigator(s):**	Prof Dr Christian Kurzeder<br>Gynäkologische Onkologie<br>Universitätsspital Basel<br>Spitalstrasse 21<br>4031 Basel<br>Email: christian.kurzeder@usb.ch<br>Phone: +41 61 265 2525<br><br>Dr med. Angela Kohler<br>Onkologie, Hämatologie & Immuntherapie<br>Kantonsspital Baselland, Liestal<br>Mühlemattstrasse 26<br>4410 Liestal<br>Email: angela.kohler@ksbl.ch<br>Phone : +41 61 925 27 28<br><br>PD Dr Marcus Vetter<br>Onkologie, Hämatologie & Immuntherapie<br>Kantonsspital Baselland, Liestal<br>Mühlemattstrasse 26<br>4410 Liestal<br>Email: marcus.vetter@ksbl.ch<br>Phone: +41 61 925 27 15<br><br>Dr med. Bich Doan Nguyen-Sträuli<br>Breast Cancer Unit<br>Klinik für Gynäkologie<br>Universitätsspital Zürich<br>Frauenklinikstrasse 10<br>8091 Zürich<br>Email: bichdoan.nguyen@usz.ch<br>Phone: +41 44 253 9676

	Dr med. Alexander Ring<br>Klinik für Medizinische Onkologie und Hämatologie<br>Universitätsspital Zürich<br>Rämistrasse 100<br>8091 Zürich<br>Email: alexander.ring@usz.ch<br>Phone: +41 43 253 0804
**Study Centre(s):**	Multi-centre
**Statistical Considerations:**	Average CTC-cluster size after treatment will be compared to average CTC-cluster size before treatment using a paired t-test. The analysis will be performed on the set of patients reaching a digoxin level of 0.7ng/ml within three cycles.
**GCP Statement:**	This study will be conducted in compliance with the protocol, the current version of the Declaration of Helsinki, the ICH-GCP or ISO EN 14155 (as far as applicable) as well as all national legal and regulatory requirements.

ABBREVIATIONS

AE	Adverse Event
BASEC	Business Administration System for Ethical Committees, (https://submissions.swissethics.ch/en/)
CA	Competent Authority (e.g. Swissmedic)
CEC	Competent Ethics Committee
CRF	Case Report Form
ClinO	Ordinance on Clinical Trials in Human Research *(in German: KlinV, in French: OClin, in Italian: OSRUm)*
eCRF	Electronic Case Report Form
CTCs	Circulating Tumor cells
CTCAE	Common terminology criteria for adverse events
DSUR	Development safety update report
GCP	Good Clinical Practice
IB	Investigator's Brochure
Ho	Null hypothesis
H1	Alternative hypothesis
HRA	Federal Act on Research involving Human Beings *(in German: HFG, in French: LRH, in Italian: LRUm)*
IMP	Investigational Medicinal Product
IIT	Investigator-initiated Trial
ISO	International Organisation for Standardisation
ITT	Intention to treat
MD	Medical Device
MedDO	Medical Device Ordinance *(in German: MepV, in French: ODim)*
PI	Principal Investigator
SDV	Source Data Verification
SOP	Standard Operating Procedure
SPC	Summary of product characteristics
SUSAR	Suspected Unexpected Serious Adverse Reaction
TMF	Trial Master File

STUDY SCHEDULE

Study Periods	Screening	Treatment, Intervention Period<br>Follow-up**				
Treatment cycle		1			2/3*	
Visit	1	2	3	4	5/7	6/8
Day	-7	0	3	7	10/17	14/21
Patient Information and Informed Consent	x					
Demographics	x					
Medical History	x					
In- /Exclusion Criteria	x					
Physical Examination	x			x		x
Vital Signs	x	x	x	x	x	x
Laboratory Tests	x			x		x
Pregnancy Test	x					
ECG	x			x		x
Digoxin serum level		x****		x		x
EDTA blood 7,5 ml for CTC isolation	x	x	x	x	x	x
Concomitant Therapy, Intervention: daily Digoxin***	x	x	x	x	x	x
Serious Adverse Events		x	x	x	x	x

* if serum digoxin level on day 7 is below 0.70 ng/ml patients will enter cycle 2 and continue digoxin maintenance treatment without dose adjustment, if serum digoxin level on day 14 is below 0.7 patients will enter cycle 3 and continue digoxin maintenance treatment with dose adjustment. To get reliable serum steady-state levels, blood should be drawn between 2 and 4 p.m. (with intake between 8 p.m.-10 p.m.)

** there is no systematic follow up, one EDTA blood sample for CTC isolation will be drawn after initiation of next cycle of systemic therapy

*** The daily dose of digoxin will be calculated according to the renal function and the target serum digoxin concentration and applied in an adjusted regimen based on the availability of 0.125 mg and 0.25 mg pills in the morning (before 10 a.m.). To get reliable serum steady-state levels, blood will be drawn in the afternoon (with intake between 8-10 am, blood sampling should be between 2-4 pm).

**** 2 hrs after first oral intake

1. STUDY ADMINISTRATIVE STRUCTURE

Steering committee: PD Dr Marcus Vetter, Prof Dr Christian Kurzeder, Prof Nicola Aceto
Cardiac safety advisor: PD Dr Gabriela Kuster-Pfister

1.1 Sponsor, Sponsor-Investigator

University Hospital Basel/ Prof Dr Christian Kurzeder

1.2 Principal Investigator(s)

Site University Hospital Basel:
Prof Dr Christian Kurzeder
University Hospital Basel
Spitalstrasse 21
4031 Basel
Phone: +41 61 26 52525
E-Mail: christian.kurzeder@usb.ch

Site Kantonsspital Baselland Liestal (KSBL)
Dr med. Angela Kohler
Kantonsspital Baselland, Liestal
Mühlemattstrasse 26
4410 Liestal
Email: angela.kohler@ksbl.ch
Tel.: +41 61 925 27 28

Site University Hospital Zurich:
Dr med. Bich Doan Nguyen-Sträuli
Breast Cancer Unit
Klinik für Gynäkologie
Universitätsspital Zürich
Frauenklinikstrasse 10
8091 Zürich
Email: bichdoan.nguyen@usz.ch
Phone: +41 44 253 9676

1.3 Statistician ("Biostatistician")

Trial Statistician:
Sabine Schädelin
Department of Clinical Research
Spitalstrasse 12
4031 Basel
Switzerland

1.4 Laboratory

Prof Dr Nicola Aceto
University of Basel
Department of Biomedicine
Cancer Metastases Laboratory

1.5 Monitoring institution

Universität Basel
Departement Klinische Forschung, On Site Management & Monitoring, Clinical Trial Unit

c/o Universitätsspital Basel
Spitalstrasse 12
4031 Basel, Switzerland

1.6 Data Safety Monitoring Committee

N/A

1.7 Any other relevant Committee, Person, Organisation, Institution

N/A

2. ETHICAL AND REGULATORY ASPECTS

Before the study will be conducted, the protocol, the proposed patient information and consent form as well as other study specific documents will be submitted to a properly constituted Competent Ethics Committee (CEC) and other Competent Authorities (CA). Any amendment to the protocol must be approved by these institutions.

The decision of the CEC and Swissmedic/foreign competent authority concerning the conduct of the study will be made in writing to the Sponsor-Investigator before commencement of this study. The clinical study can only begin once approval from all required authorities has been received. Any additional requirements imposed by the authorities shall be implemented.

2.1 Study registration

The trial will be registered before enrolment of the first patient at the U.S. National Institutes of Health clinical trial registry (clinicaltrials.gov). In addition, registration in the Swiss National Clinical trial Portal (SNCTP via BASEC) will be carried out.

2.2 Categorisation of study

The trial is categorized into risk group B. Digoxin is authorized in Switzerland for treatment of heart failure and supraventricular tachycardia. Within the trial patients with advanced or metastatic breast cancer will receive maintenance treatment with digoxin. Dosage and target serum level are in accordance with the label

2.3 Competent Ethics Committee (CEC)

The Sponsor-Investigator ensures that approval from an appropriately constituted CEC is sought for the clinical study. No changes are made to the protocol without prior Sponsor and CEC approval, except where necessary to eliminate apparent immediate hazards to study participants. Any such changes will be reported to the CEC within 7 days. Premature study end or interruption of the study is reported within 15 days. The regular end of the study is reported to the CEC within 90 days, the final study report shall be submitted within one year after study end. Amendments are reported according to chapter 2.10.

2.4 Competent Authorities (CA)

The Sponsor-Investigator will obtain approval from the CEC of 'Nordwest- and Zentralschweiz' (Leitethikkommission). The study protocol and the relevant documentation of IMP will be submitted to Swissmedic. The completion of this study will be reported to the CAs within 90 days and the final study report will be submitted within one year. In case of early discontinuation or interruption, the CA will be notified within 15 days. Non-substantial amendments will be reported as soon as possible to CAs. Substantial amendments are reported according to Section 2.10.

2.5 Ethical Conduct of the Study

The study will be carried out in accordance to the protocol and with principles enunciated in the current version of the Declaration of Helsinki, the guidelines of Good Clinical Practice (GCP) issued by ICH, in case of medical device: the European Regulation on medical devices 2017/745 and the ISO Norm 14155 and ISO 14971, the Swiss Law and Swiss regulatory authority's requirements. The CEC and regulatory authorities will receive annual safety and interim reports and be informed about study stop/end in agreement with local requirements.

2.6 Declaration of interest

The involved members of the protocol writing committee and steering committee do not have any conflicts of interest with regard to design, set-up and conduct of this study.

2.7 Patient Information and Informed Consent

The investigators will explain to each participant the nature of the study, its purpose, the procedures involved, the expected duration, the potential risks and benefits and any discomfort it may entail. Each participant will be informed that the participation in the study is voluntary and that he/she may withdraw from the study at any time and that withdrawal of consent will not affect his/her subsequent medical assistance and treatment.

The participant must be informed that his/her medical records may be examined by authorised individuals other than their treating physician.

All participants for the study will be provided a participant information sheet and a consent form describing the study and providing sufficient information for participant to make an informed decision about their participation in the study.

The formal consent of a participant, using the approved consent form, must be obtained before the participant is submitted to any study procedure.

The participant should read and consider the statement before signing and dating the informed consent form, and should be given a copy of the signed document. The consent form must also be signed and dated by the investigator (or his designee) at the same time as the participant sign, and it will be retained as part of the study records.

2.8 Participant privacy and confidentiality

The investigator affirms and upholds the principle of the participant's right to privacy and that they shall comply with applicable privacy laws. Especially, anonymity of the participants shall be guaranteed when presenting the data at scientific meetings or publishing them in scientific journals.

Individual subject medical information obtained as a result of this study is considered confidential and disclosure to third parties is prohibited. Subject confidentiality will be further ensured by utilising subject identification code numbers to correspond to treatment data in the computer files.

For data verification purposes, authorised representatives of the Sponsor (-Investigator), a competent authority (e.g. Swissmedic), or an ethics committee may require direct access to parts of the medical records relevant to the study, including participants' medical history.

2.9 Early termination of the study

The Sponsor-Investigator may terminate the study prematurely according to certain circumstances, for example:

- ethical concerns,
- insufficient participant recruitment,
- when the safety of the participants is doubtful or at risk, respectively,
- alterations in accepted clinical practice that make the continuation of a clinical trial unwise,
- early evidence of benefit or harm of the experimental intervention

2.10 Protocol amendments

Substantial amendments are only implemented after approval of the CEC and CA respectively.

Under emergency circumstances, deviations from the protocol to protect the rights, safety and well-being of human subjects may proceed without prior approval of the sponsor and the CEC/CA. Such deviations shall be documented and reported to the sponsor and the CEC/CA as soon as possible.

All non-substantial amendments are communicated to the CA as soon as possible if applicable and to the CEC within the Annual Safety Report (ASR).

3. BACKGROUND AND RATIONALE

3.1 Background and Rationale

Breast cancer (BC) is the most common invasive cancer in women, with more than one million cases and over 411,000 deaths occurring worldwide annually. Although age-adjusted mortality from breast cancer has been decreasing since 1990, the median survival for patients with metastatic disease is still only approximately 18 to 24 months and the medical need for more active agents in this clinical setting remains very high.

3.2 Investigational Product (treatment, device) and Indication

Digoxin is authorised in Switzerland for treatment of acute and chronic heart failure as well as for treatment of supraventricular tachycardia. The dosage applied is in accordance with the prescribing information

3.3 Preclinical Evidence

Circulating tumor cells are defined as those cells that depart from a primary or metastatic cancerous lesion and enter the bloodstream [Alix-Panabières C, 2014]. While extraordinarily rare compared to blood cells and forced to strive for survival in circulation, CTCs are considered to be precursors of metastasis in various cancer types, including breast cancer [Alix-Panabières C, 2014; Aceto 2015]. CTCs are found in the blood of cancer patients as single CTCs and CTC clusters [Fidler, 1973, Liotta 1976], with the latter featuring a higher ability to seed metastasis [Aceto N, 2014].

CTC cluster share several properties that commonly feature stem cell biology. Preclinical studies were able to confirm that CTC clusters differ remarkably from single CTCs from the DNA-methylation standpoint, with CTC clusters displaying hypomethylation of binding sites for a number of stemness- and proliferation-associated transcription factors including OCT4, NANOG, SOX2 and SIN3A, as well as hypermethylation of Polycomb target genes. Master stemness and proliferation regulators such as OCT4, NANOG, SOX2 and SIN3A are predominantly active in embryonic stem cells (ESCs), simultaneously regulating self-renewal and proliferation [Kim J, 2008; Masui S 2007; van den Berg DL 2010]. In addition, ESCs rely on Polycomb-mediated repression of differentiation genes and chromatin remodeling in order to maintain their active pluripotency network [Lee TI, 2006]. Importantly, cell-cell junction activity has been shown in several instances to safeguard pluripotency and to be required for a complete reprogramming of somatic cells into stem cells, and disruption of cell-cell junctions (e.g. though targeting of E-cadherin) in human ESC results into OCT4, NANOG and SOX2 downregulation along the loss of stemness features [Pieters T, 2014]. Thus, by analogy with stem cell biology, elevated expression of cell-cell junction components in cancer cells may not only enable their intravasation in the bloodstream as multicellular clusters, but also their ability to retain stem-like features that facilitate metastasis initiation.

Ouabain, digitoxin and digoxin are cardiac glycosides with a relatively similar chemical structure, acting through nonselective binding and inhibition of the Na+/K+ ATPase[11]. Upon inhibition of the Na+/K+ ATPase, a cellular uptake of Na+ occurs, leading to a simultaneous increase in intracellular Ca++ and impaired translocation of desmosomal and tight junction proteins to the cellular membrane [Altamirano J, 2006; Arispe N, 2008]. In vitro treatment of CTC-derived cell lines with ouabain and digitoxin, as well as with FCCP and CCCP compounds, support a model whereby pharmacological inhibition of the Na+/K+ ATPase either directly or indirectly (i.e. by depletion of available ATP), leads to a concomitant increase in intracellular Ca++ levels and CTC cluster disruption through suppression of functional cell-cell junction assembly [Cavey C, 2009].

Disaggregation of CTC clusters into single cells through the treatment with Na+/K+ ATPase inhibitors leads to DNA methylation remodeling at key sites and suppression of their metastatic ability. Gkountela has recently demonstrated that CTC clusters disruption into single cells via the inhibition of the Na+/K+ ATPase has a dual effect [Gkountela C, 2019]. In her studies cluster dissociation lead to DNA methylation remodeling at critical sites. Secondly, CTC clusters disruption increased the proportion of single CTCs in the bloodstream and largely suppressed overall metastasis formation in the preclinical model, indicating that targeting CTC clusters could be a valuable therapeutic strategy. Recent tumor phylogeny studies [McPherson, 2016; Reiter JG, 2017] also support the concept of anti-cluster treatment for patients with metastasis.

3.4 Clinical Evidence to Date

Digitalis compounds are among the oldest therapies for congestive heart failure and cardiac arrhythmias and still used. Their main effect is mediated through attachment to the plasma membrane Na+, K+-ATPase receptor [Wang J, 1996]. The role of digoxin as an anti-cancer drug is not established in breast cancer or other subtypes of cancer. But there are several epidemiologic studies about the use and risk of digitalis.

Cardiac glycosides as a risk factor for cancer

There is a large body of evidence that digitalis could be a risk factor for several cancers, like breast cancer [Biggar RJ, 2011], gastrointestinal cancer [Xie SH, 2017], and other cancer types. Recently a meta-analysis was published included 14 case-control studies and 15 cohort studies assessed the role of cardiac glycosides in cancer treatment [Osman MH, 2017]. All trials were published between 1976 and 2016. The conclusion of this meta-analysis was that anti-tumor activity of cardiac glycosides observed in pre-clinical studies requires high concentrations which can't be normally tolerated in humans. However, the estrogen-like activity of digitalis could be responsible for increasing the risk of certain types of tumors. Table 1 showed the outcome of epidemiologic studies.

Table 1: Outcome of digitalis use in several tumor types

Agent	Numbers	Type of Cancer	Outcome - use of digitalis	Reference
Digitoxin	9,271	Multiple	Inverse correlation for hematologic malignancies, kidney cancer and urinary tract cancer	(Haux, Klepp, Spigset, & Tretli, 2001)
Digitoxin, Digoxin and others	33	Breast	Decreased relapse rate with use	(Stenkvist et al., 1982)
Digitoxin, Digoxin and others	28	Breast	More benigne features in histology	(Stenkvist et al., 1979)
Digitoxin, Digoxin and others	175	Breast	Decreased death rate	(Stenkvist, n.d.)
Digitoxin, Digoxin and others	324	Breast	Increased risk in postmenopausal women	(Ahern, Lash, Sørensen, & Pedersen, 2008)
Digitoxin, Digoxin and others	104,648	Breast	More ER+ breast cancers	(Biggar et al., 2011)
Digoxin	145	Carcinoma	Better OS	(Menger et al., 2012)
Digoxin	786	Prostate Cancer	Decreased cancer related death	(Flahavan, Sharp, Bennett, & Barron, 2014)
Digoxin	1,006	Prostate Cancer	Inverse correlation between use and survival	(Niraula et al., n.d.)
Digoxin	47,884	Prostate Cancer	Decreased risk of death	(Platz et al.,

				2011)
Digoxin	638	Reproductive tract cancer	Reduced risk of developing uterine cancer	(Biggar, Wohlfahrt, & Melbye, 2012)

Table 1: Epidemiologic studies assessing the risk of cancer in patients using cardiac glycosides (adapted from Menger et. al. [Menger L, 2013])

Evidence for therapeutic use of cardiac glycosides in cancer

There are no randomized data available for breast cancer patients investigating the therapeutic role of digitalis glycosides. In luminal breast cancer current data suggest digitalis treatment as a risk factor. (Table 1) But data are not clear so far and the drugs have not been examined in a randomized control trial. Table 2 summarizes the current ongoing clinical trials examining digitalis in several cancer subtypes.

In 1984, Goldin et al. examined 127 patients with cancer under digitalis treatment. While patients in the control group had 21 cancer-related deaths, only one patient died in the digitalis group within in the observational period [Goldin AG, 1984].

Stenkvist et. al. published data suggesting that digitalis might have an anti-neoplastic effect in breast cancer. A cohort study with 175 patients demonstrated a better outcome if the breast cancer was diagnosed under digitalis treatment (death rate 6% vs. 34%). Also proliferation/aneuploidy was less common of the tumors in patients on digitalis [Stenkvist B, 1999].

Table 2: Clinical trials of Digitalis in several cancer types

Agent	Numbers	Type of Cancer	Outcome - use of digitalis	Reference
Anvirzel® plus Carboplatin / Docetaxel	30	NSCLC	MTD / Safety outcome	NCT01562301
Anvirzel®	18	NSCLC	MTD / Safety outcome	(Mekhail et al., 2006)
Anvirzel ®	52	Solid tumors	MTD	NCT00554268
Digoxin plus Lapatinib	17	Breast	Pharmacokinetics	NCT00650910
Digoxin plus Cisplatin/IL-2/INF-alpha2b and Vinblastin	47	Melanoma	Increased response rate from 19.5 % to 55.3%	(Atkins et al., 2008)
Digoxin plus Erlotinib	24	NSCLC	Failure to increase overall response rate	NCT00281021
Digoxin	16	Prostate Cancer	Rate of positive PSADT outcome	NCT01162135
Digoxin plus Caffeine, Midazolam, Omeprazol, s-warfarin, Vitamin K and Tivantinib	30	Solid tumors	Primary outcome : pharmakokinetics	NCT01517399
Digoxin / Capecitabine metronomic	16	Breast Cancer	Growth Modulation Index (GMI) and CBR	NCT01887288 (Haux et al., 2001)(Haux et al., 2001)

Table 2: Clinical trials using digitalis in several cancer types adapted from Menger [Menger L, 2013]

3.5 Dose Rationale

The dosage of digoxin applied is in accordance with the prescribing information. Preclinical studies could demonstrate that for digitoxin very low doses were sufficient to dissolve CTC clusters. Particularly, treatment of CTC clusters with 20nM digitoxin (a concentration that allows all cells to maintain high viability and optimal proliferation rate) was able to greatly dissociate CTC clusters and prevent metastasis formation upon injection of treated cells in animal models [Gkountela 2019].

3.6 Explanation for choice of comparator (or placebo)

As the objective is to assess the effect of digoxin on the size of CTC clusters the comparison will be within each patient. This type of comparison will allow further analyses with respect to time to response after the intervention and dose response analyses based on the serum digoxin level.

3.7 Risks / Benefits

The side effects and risks of digoxin are well known. Recent data confirmed the benefits of digoxin regarding morbidity and mortality in patients with heart failure and sinus rhythm [Guzman M, 2018]. However, contrary to the present indications for digoxin use, newer data suggest an increase in mortality in patients with atrial fibrillation [Lopez RD, 2018]. Although these findings remain somewhat controversial, patients with atrial fibrillation will be excluded from this study. Prior to treatment with digoxin patients will receive a medical examination, ECG and blood chemistry evaluation to exclude patients with contraindications. The serum level of digoxin will be evaluated every week to keep the serum concentration within the range for which mortality benefits have been shown in the cardiovascular setting and to avoid toxic plasma concentrations. Potential drug interactions with ongoing cancer treatment will be carefully evaluated before enrolment.

Patients with progressive disease will undergo routine diagnostic workup before being allocated to subsequent lines of therapy. In general study participation will be offered in the time window before start of new treatment lines. In case of clinical deterioration due to progressive disease or any need for immediate therapy, e.g. visceral crisis study treatment will be stopped if necessary (drug interactions). Any symptomatic and cancer specific treatment will therefore not be affected.

In summary the potential risk and the side effects of the IMP have been extensively studied and patients at risk will not be included. Only patients with advanced disease stages and no curative treatment option will be included. Based on preclinical data there is a strong rationale for the use of digoxin in breast cancer patients and observational trials have shown that cardiac glycosides could potentially improve the prognosis of cancer patients.

3.8 Justification of choice of study population

The prognosis of patients included in the study is still poor. CTC clusters have been detected with higher frequency in patients with advanced or metastasized breast cancer as compared with early disease stage patients. The goal of this exploratory trial is to proof the concept that CTC cluster can be disrupted by treatment with cardiac glycosides. As hypothesized, a major clinical effect of CTC cluster disruption could be the prevention of metastasis formation in early breast cancer patients. However, for a variety of advanced stage cancer models, beneficial effects have been postulated based on the suppression of the metastatic potential of CTC clusters, especially in those cases where metastatic foci can spread and give rise to additional metastases.

4. STUDY OBJECTIVES

4.1 Overall Objective

The overall objective is to evaluate whether cardiac glycosides are able to disrupt CTC clusters in breast cancer patients as shown in preclinical models.

4.2 Primary Objective

The primary objective is to assess the effect of digoxin on the size of CTC clusters in patients with advanced breast cancer

4.3 Secondary Objectives

The secondary objectives are to examine the effect of digoxin on the number of CTC clusters detected in patients with advanced breast cancer and to investigate the kinetics of dissolution of CTC clusters in vivo. Furthermore the dose response relationship of the effect shall be explored.

4.4 Safety Objectives

N/A

5. STUDY OUTCOMES

5.1 Primary Outcome

The primary study outcome is the mean CTC cluster size in patients with a digoxin serum level above 0.7 ng/ml

5.2 Secondary Outcomes

Secondary study outcomes include the mean CTC cluster number, average time to dissolution of CTC clusters

5.3 Other Outcomes of Interest

As an exploratory outcome mean CTC cluster size after initiation of anticancer therapy will be recorded

5.4 Safety Outcomes

N/A

6. STUDY DESIGN

6.1 General study design and justification of design

This will be a single arm therapeutic exploratory study of digoxin in patients with advanced or metastatic breast cancer and circulating tumor cell clusters.

Patients will first be screened for the presence of CTC clusters in their blood. Only patients in whom at least one cluster of CTCs is detectable at screening will enter treatment phase.

Patients with advanced or metastatic breast cancer in whom CTC clusters could be identified will receive a daily maintenance dose of digoxin. Blood samples for analyses of digoxin serum level and mean CTC cluster size will be drawn at specified time points.

To determine the effect of digoxin on CTC cluster size 9 patients with Digoxin level within target range will be included and blood samples for isolation of CTC clusters will be drawn before initiation of digoxin treatment and at specific time points after initiation of therapy.

Assuming that CTC clusters can be detected in 20-25% of eligible patients, 50-60 patients need to be screened.

After stop of IMP administration a follow up visit will be scheduled to draw one further blood sample after initiation of the next line of systemic therapy.

6.2 Methods of minimising bias

6.2.1 Randomisation

N/A

6.2.2 Blinding procedures

N/A

6.2.3 Other methods of minimising bias

N/A

6.3 Unblinding Procedures (Code break)

N/A

7. STUDY POPULATION

7.1 Eligibility criteria

Inclusion criteria:

- Informed Consent as documented by signature
- Adult women and men (≥ 18 years of age) with proven diagnosis of adenocarcinoma of the breast with evidence of locoregionally recurrent or metastatic disease not amenable to resection, radiation therapy or systemic therapy with curative intent
- Adequate organ and marrow function defined as follows:
- ANC ≥1,500/mm3 (1.5 x 109 /L);
- Platelets ≥100,000/mm3 (100 x 109 /L);
- Hemoglobin ≥9 g/dL (90 g/L);
- Serum creatinine ≤1.5 x ULN or estimated creatinine clearance ≥ 60 mL/min as calculated using the method standard for the institution;
- Total serum bilirubin ≤1.5 x ULN (≤3.0 x ULN if Gilbert's disease);
- AST and/or ALT ≤3 x ULN (≤5.0 x ULN if liver metastases present);
- Alkaline phosphatase ≤2.5 x ULN (≤5.0 x ULN if bone or liver metastases present).

- Resolution of all acute toxic effects of prior anti-cancer therapy or surgical procedures to NCI CTCAE version 4.0 Grade ≤1 (except alopecia or other toxicities not considered a safety risk for the patient at investigator's discretion).

Exclusion:

- Patients on treatment with digoxin or digitoxin
- Patients with atrial fibrillation or atrial flutter
- Ventricular Fibrillation or ventricular tachycardia,
- Atrioventricular heart block 2nd or 3rd degree, sick sinus syndrome or sinus bradycardia,
- Wolff-Parkinson-White Syndromeor
- Hypokalemia, hypercalcemia, hypomagnesemia,
- Hypoxia,
- hypertrophic cardiomyopathy,
- aortic aneurysm,
- simultaneous intravenous application of calcium salts
- Known hypersensitivity to Digoxin, other cardiac glycosides or included compounds
- Known drug interactions of ongoing cancer therapy with digoxin
- Women who are pregnant or breast feeding,
- Intention to become pregnant during the course of the study,
- Lack of safe contraception, defined as: Female participants of childbearing potential, not using and not willing to continue using a medically reliable method of contraception for the entire study duration, such as oral, injectable, or implantable contraceptives, or intrauterine contraceptive devices, or who are not using any other method considered sufficiently reliable by the investigator in individual cases.
- Female participants who are surgically sterilised / hysterectomised or post-menopausal for longer than 2 years are not considered as being of child bearing potential.
- Other clinically significant concomitant disease states (e.g., renal failure, hepatic dysfunction, cardiovascular disease, etc.),
- Known or suspected non-compliance, drug or alcohol abuse,
- Inability to follow the procedures of the study, e.g. due to language problems, psychological disorders, dementia, etc. of the participant,
- Participation in another study with investigational drug within the 30 days preceding and during the present study,
- Previous enrolment into the current study,
- Enrolment of the investigator, his/her family members, employees and other dependent persons,

7.2 Recruitment and screening

Patients will be screened and recruited by the treating oncologist. There will be no financial compensation to study participants.

7.3 Assignment to study groups

N/A

7.4 Criteria for withdrawal / discontinuation of participants

Patients have the right to refuse further treatment for any reason and at any time. For the patient’s security, a last examination should be performed. Patients may be withdrawn at any time from trial treatment at the discretion of the investigator due to a SAE, or based on any other relevant medical condition.

8. STUDY INTERVENTION

8.1 Identity of Investigational Products (treatment / medical device)

Patients will receive oral treatment with digoxin in accordance with the prescribing information

8.1.1 Experimental Intervention (treatment / medical device)

Digoxin is licenced in Switzerland and will be bought at pharmacy and labelled as study medication.

8.1.2 Control Intervention (standard/routine/comparator treatment / medical device)

N/A.

8.1.3 Packaging, Labelling and Supply (re-supply)

N/A

8.1.4 Storage Conditions

N/A

8.2 Administration of experimental and control interventions

8.2.1 Experimental Intervention

Patients will receive a daily maintenance dose of digoxin as calculated based on renal function and target serum concentration [Muzarelli S, 2010]. The calculated daily dose will be applied in an adjusted regimen based on the availability of 0.125 and 0.25 mg pills in the morning (before 10 am and ideally at the same time each day). Blood samples for analyses of mean CTC cluster size will be drawn on day 0 (2 hrs after first oral intake), on day 3 and on day 7. Depending on the digoxin serum level (as measured in the afternoon to be as close as possible to steady-state levels) maintenance therapy with digoxin will be continued up to 3 weeks if the digoxin serum level on day 7 or day 14 is below 0.70 ng/ml. For the third week of maintenance therapy individual dose adjustments will be carried out.

8.2.2 Control Intervention

N/A

8.3 Dose / Device modifications

Digoxin pills of 0.125 and 0.25 will be bought from pharmacy stocks and labelled as study medication. The calculated dose of digoxin will be maintained for 1 week, after which serum digoxin concentrations will be measured. In case the lower target concentration of 0.7 ng/ml will not be reached, the same dose will be applied for another week. Only when serum digoxin concentrations remain too low after 2 weeks, the daily dose of digoxin will be increased. In case the serum digoxin concentration lies above the upper target concentration (1.0 ng/ml) the dose of digoxin will be immediately reduced (after week 1 and/or 2).

8.4 Compliance with study intervention

The digoxin serum level will be determined weekly.

8.5 Data Collection and Follow-up for withdrawn participants

Patients withdrawing from study will have a final safety visit. Data obtained during study until withdrawal will be used for analysis unless the patient clearly declines use of data collected in study. After withdrawal from study no further follow-up will be conducted. No blood samples of pateints will be stored.

8.6 Trial specific preventive measures

Treatments listed below are not permitted during the trial treatment phase. If they are medically required during treatment phase, the patient will be directly transferred to the follow-up phase.

- Calcium if administered i.v.
- Drugs which could affect serum electrolyte levels such as diuretics, Laxantien, Benzylpenicillin, Amphotericin B, corticosteroids, ACTH, Salicylate, Lithiumsalze
- Beta-adrenergic blockers , additive effects on AV node conduction can result in bradycardia and advanced or complete heart block
- drugs that induce/inhibit P-glycoprotein have the potential to alter digoxin pharmacokinetics and are contraindicated: Atorvastatin, calcium channel blockers (i.e. Verapamil, Felodipin), Captopril, Spironolacton, Itraconazol, Chinin, Atropin, Ciclosporin, Ritonavir and Saquinavir, Antiarrhythmics (Chinidin, Amiodaron, Flecainid, Propafenon), Indomethacin and Alprazolam
- drugs that could effect the metabolism of digoxin such as Makrolidantibiotics [Clarithromycin, Erythromycin und Azithromycin], Tetracycline, Gentamicin, Trimethoprim
- drugs which could cause cardiac arrhythmia such as Suxamethoniumchlorid, Reserpin, tricyclic antidepressive agents, sympathomimetics, phosphodiesterase inhibitors (i.e. Theophyllin)
- Loperamid

To rule out cardiac arrhythmia which would not allow to begin or proceed with Digoxin treatment all patients are required to receive an ECG at screening. If treatment is to be continued for a second or third week the ECG will be repeated.

8.7 Concomitant Interventions (treatments)

All drugs that are not listed in 8.6 are in principal allowed, but potential drug interactions will be assessed using online tools before enrolment (www.compendium.ch; “Interaktionen” via GLN login). A new line of anticancer therapy should be started after stop of IMP intake.

8.8 Study Drug / Medical Device Accountability

The local investigators will be responsible for adequate labelling and storage of the IMP at the hospital pharmacy or in the department. The Principal Investigator of the individual site must maintain a careful record of the inventory and disposition of all study agents received, using the Drug Accountability Record Form (DARF).

8.9 Return or Destruction of Study Drug / Medical Device

Study agents not distributed will be returned to hospital pharmacy for destruction.

9. STUDY ASSESSMENTS

9.1 Study flow chart(s) / table of study procedures and assessments

Planned duration of treatment is 1 week. Depending on the digoxin serum level after each cycle of treatment maintenance therapy with digoxin will be continued up to 3 weeks if the digoxin serum level on day 7 or day 14 is below 0.70 ng/ml. For the third week of maintenance therapy individual dose adjustments will be carried out.

In case one of the following events occurs during the treatment phase, IMP treatment will be terminated:

- unacceptable toxicity
- patient refusal
- withdrawal by the physician
- start of a not permitted treatment

9.2 Assessments of outcomes

9.2.1 Assessment of primary outcome

EDTA blood (7.5 ml each) for CTC isolation will be drawn for analyses of the primary outcome

9.2.2 Assessment of secondary outcomes

See also 9.2.1

9.2.3 Assessment of other outcomes of interest

EDTA blood for CTC isolation will be drawn for analyses of the primary outcome

9.2.4 Assessment of safety outcomes

9.2.4.1 Adverse events

Regarding adverse events, we will collect: time of onset, duration, resolution, action been taken,

assessment of intensity, relationship with study treatment (see section 10) at each treatment visit every 28 days and at any time at acute onset.

9.2.4.2 Laboratory parameters

Laboratory parameters including a full blood count, serum electrolytes, liver function and kidney function will be captured after each weekly cycle of treatment.

9.2.4.3 Vital signs

Vital signs such as body temperature, blood pressure and heartbeat will be assessed at each treatment visit. . In addition, at each week after initiation of digoxin therapy, serum digoxin levels will be determined.

9.2.4.4 ECG

An ECG will be carried out at screening and at each weekly cycle of digoxin treatment, and at any time adverse events and/or abnormalities in serum electrolytes will be detected

9.2.5 Assessments in participants who prematurely stop the study

Adverse events, laboratory parameters and vital signs will be recorded

9.3 Procedures at each visit

9.3.1 Screening visit

To be performed within 7 days before registration

- Patient Information and Informed Consent
- Demographics
- Medical History baseline symptoms, previous therapies, location of tumor sites
- Documentation of most recent Tumor assessments by CT or MRI or PET CT imaging
- Concomitant Therapy
- In- /Exclusion Criteria
- Vital signs and physical measurements (blood pressure, heart rate, height, weight, ECOG
- performance status and physical examination)
- Laboratory Tests:
  - Haematology: haemoglobin, neutrophils, platelets
  - Biochemistry: calcium, potassium, sodium, magnesium
  - Hepatic function: AP, AST, ALT, bilirubin
  - Renal function: serum creatinine, calculated creatinine clearance (according to the formula of Cockcroft-Gault)
- Urine pregnancy test for women with child-bearing potential
- ECG
- EDTA blood for CTC isolation

9.3.2 Visit 2, 3, 5, 7

To be performed during the treatment phase

- Vital Signs
- EDTA blood for CTC isolation (7.5 ml)
- Concomitant Therapy, Intervention
- Adverse Events
- Only visit 2: Digoxin serum level 2 hrs after first oral intake

9.3.3 Visit 4, 6, 8

To be performed during the treatment phase

- Physical Examination
- Vital Signs
- Laboratory Tests
- Digoxin serum level
- EDTA blood for CTC isolation (7.5 ml)
- Concomitant Therapy
- Adverse Events
- ECG

10. SAFETY

10.1 Drug studies

The Sponsor's SOPs provide more detail on safety reporting.

During the entire duration of the study, all serious adverse events (SAEs) are collected, fully investigated and documented in source documents and case report forms (CRF)) in SecuTrial®.. Study duration encompassed the time from when the participant signs the informed consent until the last protocol-specific procedure has been completed, including a safety follow-up period.

10.1.1 Definition and assessment of (serious) adverse events and other safety related events

An **Adverse Event (AE)** is any untoward medical occurrence in a patient or a clinical investigation participant administered a pharmaceutical product and which does not necessarily have a causal relationship with the study procedure. An AE can therefore be any unfavourable and unintended sign (including an abnormal laboratory finding), symptom, or disease temporally associated with the use of a medicinal (investigational) product, whether or not related to the medicinal (investigational) product. [ICH E6 1.2]

A **Serious Adverse Event (SAE)** is classified as any untoward medical occurrence that:

- results in death,
- is life-threatening,
- requires in-patient hospitalization or prolongation of existing hospitalisation,
- results in persistent or significant disability/incapacity, or
- is a congenital anomaly/birth defect.

In addition, important medical events that may not be immediately life-threatening or result in death, or require hospitalisation, but may jeopardise the patient or may require intervention to prevent one of the other outcomes listed above should also usually be considered serious. [ICH E2A]

SAEs should be followed until resolution or stabilisation. Participants with ongoing SAEs at study termination (including safety visit) will be further followed up until recovery or until stabilisation of the disease after termination.

Assessment of Causality

Both Investigator and Sponsor-investigator make a causality assessment of the event to the study drug, based on the criteria listed in the ICH E2A guidelines:

Relationship	Description
Definitely	Temporal relationship<br>Improvement after dechallenge*<br>Recurrence after rechallenge<br>(or other proof of drug cause)
Probably	Temporal relationship<br>Improvement after dechallenge<br>No other cause evident
Possibly	Temporal relationship<br>Other cause possible
Unlikely	Any assessable reaction that does not fulfil the above conditions
Not related	Causal relationship can be ruled out
*Improvement after dechallenge only taken into consideration, if applicable to reaction	

Unexpected Adverse Drug Reaction

An "unexpected" adverse drug reaction is an adverse reaction, the nature or severity of which is not consistent with the applicable product information (e.g. Investigator's Brochure for drugs that are not yet

approved and Product Information for approved drugs, respectively). [ICH E2A]

Suspected Unexpected Serious Adverse Reactions (SUSARs)

The Sponsor-Investigator evaluates any SAE that has been reported regarding seriousness, causality and expectedness. If the event is related to the investigational product and is both serious and unexpected, it is classified as a SUSAR.

Assessment of Severity

AEs are coded with the NCI Common Terminology Criteria for Adverse Events (CTCAE) v4.0, and assigned a grade (from 1 = mild to 5 = death related to AE) as well as a relationship to trial treatment. The NCI CTCAE v4.0 (as pdf) as well as instructions on how to use the criteria can be found on http://ctep.cancer.gov/protocolDevelopment/electronic_applications/ctc.htm.

10.1.2 Reporting of serious adverse events (SAE) and other safety related events

Reporting of SAEs

All SAEs must be reported immediately and within a maximum of 24 hours to the Sponsor-Investigator of the study. The Sponsor-Investigator will re-evaluate the SAE and return the form to the site.

SAEs resulting in death are reported to the Ethics Committee via BASEC within 7 days.

Exemptions from expedited reporting may include SAEs which are a clear result of the underlying disease.

Reporting of SUSARs

A SUSAR needs to be reported to the Ethics Committee (local event via local Investigator) via BASEC and to Swissmedic for category B and C studies (via Sponsor-Investigator) within 7 days, if the event is fatal, or within 15 days (all other events).

Reporting of Safety Signals

All suspected new risks and relevant new aspects of known adverse reactions that require safety-related measures, i.e. so called safety signals, must be reported to the Sponsor-Investigator within 24 hours. The Sponsor-Investigator must report the safety signals within 7 days to the Ethics Committee (local event via local Investigator) via BASEC and to Swissmedic in case of a category B or C study.

Reporting and Handling of Pregnancies

Pregnant participants must immediately be withdrawn from the clinical study. Any pregnancy during the treatment phase of the study and within 30 days after discontinuation of study medication will be reported to the Sponsor-Investigator within 24 hours. The course and outcome of the pregnancy should be followed up carefully, and any abnormal outcome regarding the mother or the child should be documented and reported.

Periodic reporting of safety

An annual safety report is submitted once a year to the local Ethics Committee via local Investigator and to Swissmedic in case of a category B or C study via Sponsor-Investigator.

10.1.3 Follow up of (Serious) Adverse Events

In case patients experience an SAE and are withdrawn from the study, the SAE will be followed-up until resolved and reported as if the patient was on study. Only in case the patient declines further reporting to the Sponsor the follow-up of SAE will not be reported. However, the treating oncologist will be encouraged to tightly follow-up the patient as per local practise and document the progress in the local health record system.

10.2 Medical Device Category C studies

N/A

10.3 Medical Device Category A studies

N/A

10.4 Assessment, notification and reporting on the use of radiation sources

N/A

11. STATISTICAL METHODS

11.1 Hypothesis

H0: In patients who reached a digoxin level of 0.7 ng/ml within the three treatment cycles, the average CTC cluster size after digoxin treatment is not smaller. Compared to before treatment.

H1: In patients who reached a digoxin level of 0.7 ng/ml within the three treatment cycles, the average CTC cluster size after digoxin treatment is smaller compared to before treatment.

11.2 Determination of Sample Size

This study is powered for the number of patients with CTC-clusters who reach the Digoxin target level within 3 weeks after onset of treatment, i.e. the digoxin efficacy set (DES). Patients will be included and scanned for CTC-clusters until the required number of patients qualify for the Digoxin treatment and reach the target level.

It is expected that only 20-25 % of all patients in the study population have CTC-clusters. Furthermore it is expected that 20-25% of the patients don't reach the Digoxin target level. Therefore the total number of patients included in the full analysis set (FAS) will be around 50-60. The sample size estimation was based on pilot data. The comparison will be within each patient, therefore a paired test will be used. Since this is a phase II study and the digoxin treatment will be further developed if and only if there is some evidence for its efficacy in resolving the CTC-clusters, the tests will be one-sided. Each sample size, ni=1,...,19 = 2, ..., 20, was evaluated assuming a correlation of the average cluster size before and after treatment of 0.5. For each sample size the power to show a significant reduction of the average CTC cluster size was assessed in a paired t-test assuming a mean reduction of 1.1. Thereby a one-sided alpha-level of 0.05 was used. For this study, 12 patients should be recruited to ensure 9 evaluable patients, assuming that 20 % of the patients do not reach the target level within three cycles and therefore are not part of the main analysis set (DES). This sample size allows with a power of 0.8 to estimate a mean treatment effect of digoxin of 1.1 (average CTC-cluster size reduction in ng/ml).

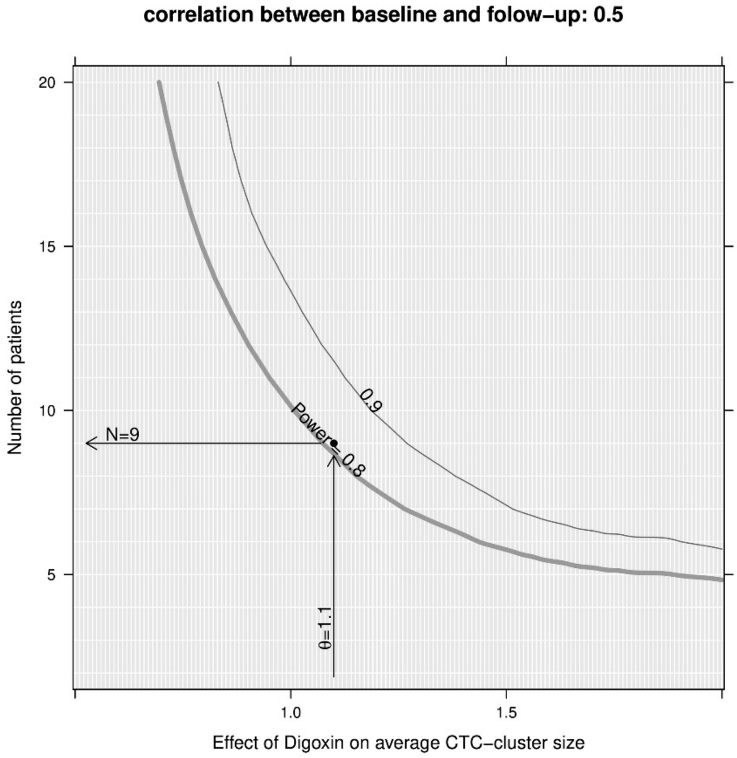

Figure 1: Sensitivity of the sample size with respect to the expected treatment effect of digoxin. The curves for a power of 0.8 and 0.9 (ie. 80 % and 90 %) are shown. (The curves are smoothed and are shown for illustration only)

11.3 Statistical criteria of termination of trial

N/A

11.4 Planned Analyses

11.4.1 Datasets to be analysed, analysis populations

The full analysis data set (FAS) consists of all patients included in the trial. The treated patients set (TPS) includes all patients who have at least one CTC-cluster at baseline and therefore qualify for digoxin treatment. The digoxin efficacy set (DES) consists of patients who reach a digoxin level of 0.7 (in ng/ml) within three cycles.

11.4.2 Demographic and baseline characteristics

Demographics and relevant baseline variables will be summarized for the FAS and the DES data set. For the FAS, the data will be stratified by CTC-clusters at baseline (ie. part of the TPS vs. not part of the TPS). Categorical data will be presented as frequencies and percentages. For continuous variables, the mean and the standard deviation or the median and the first and third quartile will be presented as appropriate.

11.4.3 Primary Analysis

Average CTC-cluster size after treatment will be compared to average CTC-cluster size before treatment using a paired t-test. The analysis will be performed on the DES set. The analysis will be performed on complete cases. However for patients with no clusters after digoxin treatment, average cluster size 1 will be imputed assuming that all clusters dissolved. No further imputations for missing values will be used.

Sensitivity analysis: Given the low number of patients the normality of the beforeafter treatment change can not be assessed reliably. Therefore the hypothesis will also be assessed in a permutation test, void of underlying statistical assumptions with regards to the distribution. To this end, the within-patient order of the measurements (“average CTC-cluster size before treatment” vs “average CTC-cluster size after treatment”) will be randomly shuffled and the t-value used in the paired t-test will be estimated. This process will be repeated 999 times. The obtained t-values will be ordered and it will be assessed, if the observed t-value when using the unshuffled data is contained within the lower 95 % (100-α, one-sided) of them. If not, the null hypothesis will be rejected.

11.4.4 Secondary Analyses

The secondary objective is to investigate potential effects of digoxin on the number of clusters and the time to dissolution of CTC-clusters. The number of CTC-clusters before and after treatment will be compared using a permutation test as described for the main analysis (sensitivity analysis). Furthermore the average CTC-cluster size vs. number of clusters before and after treatment will be presented graphically. The dose response will be investigated be color-coding this figure according to the digoxin level reached. The size of each CTC-cluster will be presented descriptively before and after treatment in the TPS. Time to the point patients reached the digoxin target level and the average CTC-cluster size at each blood sample will be summarized. For the FAS, the proportion of patients without CTC-clusters will be reported.

11.4.5 Interim analyses

No interim analyses is planned.

11.4.6 Safety analysis

Safety will be assessed by rigorous examination and detailed presentation of severe adverse events.

11.4.7 Deviation(s) from the original statistical plan

If substantial deviations of the analysis as outlined in these sections are needed for whatever reason, the protocol will be amended. All deviations of the analysis from the protocol or from the detailed analysis plan will be listed and justified in a separate section of the final statistical report.

11.5 Handling of missing data and drop-outs

Careful trial planning and conducting will minimize the occurrence of missing data as far as possible. No imputation will be performed except for average CTC-cluster size = 1 in patients who do not have

any further clusters after digoxin treatment.

12. QUALITY ASSURANCE AND CONTROL

The monitor (Clinical Trial Unit at the University Hospital Basel) will conduct a study initiation visit to verify the qualifications of the local investigator, inspect the facilities, and inform the Investigator of responsibilities and procedures for ensuring adequate and correct documentation. The Investigator must prepare and maintain adequate and accurate records of all observations and other data pertinent to the clinical study for each study participant. The Investigator will make all appropriate safety assessments on an ongoing basis.

All aspects of the study will be carefully monitored with respect to Good Clinical Practices (GCP) and standard operating procedures (SOPs) for compliance with applicable government regulations. The Study Monitor will be an authorized individual designated by the Sponsor. The Study Monitor will have access to all records necessary to ensure integrity of the data and will periodically review the progress of the study with the PI via secuTrial®.

12.1 Data handling and record keeping / archiving

The investigator must maintain adequate and accurate records to enable the conduct of the clinical trial to be fully documented and the trial data to be subsequently verified. These documents should be classified into two different categories: (A) Investigators Site File and (B) patient clinical source documents.

The study will use an electronic case report form (eCRF named secuTrial®) for data collection and documentation, which is hosted by the Clinical Trial Unit (CTU) of the University Hospital Basel. The data are entered directly via web browser to the eCRF and are transferred via encryption to the central database.

Before a user gets access to the productive environment, the user account is only activated for training. After the user has activated its account the user management at CTU enables the user for the appropriate site. The access level depends on the group membership (investigator, study nurse, monitor etc.). Users with monitoring function only are not able to enter or change patient's data. Users of SecuTrial receive training material (secuTrial® Manual), which is provided by the CTU Basel.

12.1.1 Case Report Forms

Source data need to be collected safely by authorized personnel at the site (digital or in paper) and made be available for the monitor. The completed eCRF must be electronically signed (authorization) at the end of each visit for each patient by the local/site investigator. In order to ensure the anonymity of the patient data, the data are recorded by number. An allocation list, containing the patient identifier, is kept at the local site only. If a patient is withdrawn, every effort should be made to collect follow up data.

12.1.2 Specification of source documents

Source data in this trial are laboratory, examination, tumor marker or pathology results, vital signs, radiological reports, pregnancy test, concomitant medication, medical history, survival status

12.1.3 Record keeping / archiving

In a multistage procedure, the given data will be checked electronically for their plausibility and consistency. Even during data collection, implausible data will be flagged automatically by implemented validation checks. Detected inconsistencies and missing or implausible data will be clarified with queries (electronically or paper-based) and necessary changes will be carried out. secuTrial® system has an implemented audit trial. Storage of data will be up to 20 years.

12.2 Data management

12.2.1 Data Management System

The clinical trial data will be collected anonymously in an electronic data capture system, named secuTrial®. secuTrial® runs on a server maintained by the IT-Department of the University Hospital Basel. It is implemented (set-up and adjusted) by the data management group at the Clinical Trial Unit

at the University Hospital Basel. Data management at the Clinical Trial Unit of the University Hospital of Basel will be performed as to their standard operating procedures, see CDMA Planning (2.0.1), CDMA Development, Testing and Release (2.0.1), CDMA Training (1.0.1), CDMA Locking and Closure (1.1.0). Each study site is responsible for data entry into the secuTrial® system.

12.2.2 Data security, access and back-up

The secuTrial® system is accessible via a standard browser on a WWW-connected device. Password protection ensures that only authorized persons can enter the system to view, add or edit data according to their permissions. User administration and user training is performed by the Clinical Trial Unit of the University Hospital Basel according to predefined processes. Back-up of secuTrial® study data is performed according to the processes of the IT-Department of the University Hospital Basel.

12.2.3 Analysis and archiving

The secuTrial® database will be locked after all data was monitored and all raised queries have been resolved. Data is exported and transferred to the investigator by the Clinical Trial Unit University Hospital Basel according to internally defined processes. Data will be archived by the investigator

12.2.4 Electronic and central data validation

Data is entered into the eCRF (secuTrial®) and can be validated for completeness and discrepancies automatically. An audit trail system maintains a record of initial entries and changes (reasons for changes, time and date of changes, user identification of entry and changes).
At all times, the Investigator has final responsibility for the accuracy and authenticity of all clinical data. The data entered into the eCRF will be reviewed by the responsible investigator and an independent monitor will raise queries using the query management system implemented. Designated investigators have to respond to the query and confirm or correct the corresponding data. Thereafter the monitor can close the query.

12.3 Monitoring

A monitor from the Clinical Trial Unit of the University of Basel will contact and visit all sites regularly. The monitor will verify the adherence to the protocol and the completeness, consistence and accuracy of the data being entered on eCRF, that the study is being conducted according to the protocol and within the specified time frame and that the facilities and staff remain adequate.

The monitor will require access to all patient medical records including laboratory test results and surgery, pathology and radiology reports and supporting documents to verify the entries on the eCRF. The investigator (or his/her designee) should work with the monitor to ensure that any problems detected during these visits are resolved and ensure that source data and documents are made accessible to the Study Monitor and answer questions by the Study Monitor. Risk Based Quality Management and Risk Based Monitoring will apply according to the monitoring plan, defined from CTU Basel.

12.4 Audits and Inspections

Authorities have the right to perform inspections, and the Sponsor has the right to perform on-site auditing during working hours upon reasonable prior notice.

12.5 Confidentiality, Data Protection

Data generation, transmission, archiving and analysis of health related personal data and the storage of biological samples within this project strictly follow the current Swiss legal requirements for data protection and according to the Ordinance HFV Art. 5. Prerequisite is the voluntary approval of the participant given by signing the informed consent prior start of participation of the research project. Outside the project site, confidentiality will be ensured by encoding sample and data. The code will be protected against unauthorized access and will be stored appropriately by the project leaders.

A unique patient number will be attributed to each patient registered into the project. The UPN will be the tracking number for the biopsy and all subsequent analyses. Identification of patients will only be possible at the site, not in any external laboratory conducting the experiments. For this purpose

specific patient screening and enrolment and patient identification lists for the project are to be used. To avoid identification errors, the year of birth and the UPN must be provided on the eCRFs.

12.6 Storage of biological material and related health data

N/A

13. PUBLICATION AND DISSEMINATION POLICY

Results of this study will be published irrespective of the nature of findings. Members of the steering committee are primarily responsible to analyse the data, draft the manuscript and submit the work for publication to a peer-reviewed medical journal.

14. FUNDING AND SUPPORT

14.1 Funding

The trial will be funded by research funds of the University Hospital Basel and by research funds of Prof Nicola Aceto.

14.2 Other Support

N/A

15. INSURANCE

Insurance will be provided by the Sponsor. A copy of the certificate is filed in each investigator site file and the trial master file.

The sponsor-project leader will allow delegates of the insurance company to have access to the source data / documents as necessary to clarify a case of damage related to project participation. In this context, all involved parties will keep the participants'' data strictly confidential.

16. REFERENCES

1. Declaration of Helsinki, Version October 2013, (http://www.wma.net/en/30publications/10policies/b3/index.html)
2. International Conference on Harmonization (ICH, 1996) E6 Guideline for Good Clinical Practice. (http://www.ich.org/fileadmin/Public_Web_Site/ICH_Products/Guidelines/Efficacy/E6/E6_R2__Step_4.pdf)
3. International Conference on Harmonization (ICH, 1997) E8 Guideline: General Considerations for Clinical Trials http://www.ich.org/fileadmin/Public_Web_Site/ICH_Products/Guidelines/Efficacy/E8/Step4/E8_Guideline.pdf)
4. Humanforschungsgesetz, HFG Bundesgesetz über die Forschung am Menschen (Bundesgesetz über die Forschung am Menschen, HFG) vom 30. September 2011/ Loi fédérale relative à la recherche sur l’être humain (loi relative à la recherche sur l’être humain, LRH) du 30 septembre 2011 / Legge federale concernente la ricerca sull'essere umano (Legge sulla ricerca umana, LRUm) del 30 settembre 2011
5. Verordnung über klinische Versuche in der Humanforschung (Verordnung über klinische Versuche, KlinV) vom 20. September 2013 / Ordonnance sur les essais cliniques dans le cadre de la recherche sur l’être humain (Ordonnance sur les essais cliniques, OClin) du 20 septembre 2013. Ordinanza sulle sperimentazioni cliniche nella ricerca umana (Ordinanza sulle sperimentazioni cliniche, OSRUm) del 20 settembre 2013
6. Heilmittelgesetz, HMG Bundesgesetz über Arzneimittel und Medizinprodukte (Heilmittelgesetz, HMG) vom 15. Dezember 2000 / Loi fédérale sur les médicaments et les dispositifs médicaux (Loi sur les produits thérapeutiques, LPT) du 15 décembre 2000 / Legge federale sui medicamenti e i dispositivi medici (Legge sugli agenti terapeutici, LATer)
7. ISO 14155:2011 Clinical investigation of medical devices for human subjects -- Good clinical practice (www.iso.org)
8. ISO 10993 Biological evaluation of medical devices (www.iso.org)
9. MEDDEV 2.7/3 revision 3, May 2015
10. Medizinprodukteverordnung (MepV) vom 17. Oktober 2001 / Ordonnance sur les dispositifs médicaux (ODim) du 17 octobre 2001 / Ordinanza relativa ai dispositivi medici (ODmed) del 17 ottobre 2001
11. WHO, International Clinical Trials Registry Platform (ICTRP) (http://www.who.int/ictrp/en/)
12. European regulation on medical devices 2017/745.
13. Alix-Panabières C, Pantel K. Nat Rev Cancer. 2014 Sep;14(9):623-31. doi: 10.1038/nrc3820. Epub 2014 Jul 31. Review.
14. Aceto N, Toner M, Maheswaran S, Haber DA Trends Cancer. 2015 Sep;1(1):44-52. doi: 10.1016/j.trecan.2015.07.006. Epub 2015 Sep 28. Review.
15. Fidler IJ. The relationship of embolic homogeneity, number, size and viability to the incidence of experimental metastasis. Eur J Cancer. 1973 Mar;9(3):223-7.
16. Liotta LA, Saidel MG, Kleinerman J, The significance of hematogenous tumor cell clumps in the metastatic process. Cancer Res. 1976 Mar;36(3):889-94.
17. Aceto N, Bardia A, Miyamoto DT, Donaldson MC, Wittner BS, Spencer JA, Yu M, Pely A, Engstrom A, Zhu H, Brannigan BW, Kapur R, Stott SL, Shioda T, Ramaswamy S, Ting DT, Lin CP, Toner M, Haber DA, Maheswaran S. Circulating tumor cell clusters are oligoclonal precursors of breast cancer metastasis. Cell. 2014 Aug 28;158(5):1110-1122. doi: 10.1016/j.cell.2014.07.013
18. Kim J, Chu J, Shen X, Wang J, Orkin SH. An extended transcriptional network for pluripotency of embryonic stem cells. Cell. 2008 Mar 21;132(6):1049-61. doi: 10.1016/j.cell.2008.02.039. Erratum in: Cell. 2008 Jun 27;133(7):1290
19. Masui S, Nakatake Y, Toyooka Y, Shimosato D, Yagi R, Takahashi K, Okochi H, Okuda A, Matoba R, Sharov AA, Ko MS, Niwa H. Pluripotency governed by Sox2 via regulation of Oct3/4 expression in mouse embryonic stem cells. Nat Cell Biol. 2007 Jun;9(6):625-35. Epub 2007 May 21.
20. van den Berg DL, Snoek T, Mullin NP, Yates A, Bezstarosti K, Demmers J, Chambers I, Poot RA. An Oct4-centered protein interaction network in embryonic stem cells. Cell Stem Cell. 2010 Apr 2;6(4):369-81. doi: 10.1016/j.stem.2010.02.014.
21. Lee TI, Jenner RG, Boyer LA, Guenther MG, Levine SS, Kumar RM, Chevalier B, Johnstone SE, Cole MF, Isono K, Koseki H, Fuchikami T, Abe K, Murray HL, Zucker JP, Yuan B, Bell GW, Herbolsheimer E, Hannett NM, Sun K, Odom DT, Otte AP, Volkert TL, Bartel DP, Melton

DA, Gifford DK, Jaenisch R, Young RA. Control of developmental regulators by Polycomb in human embryonic stem cells. Cell. 2006 Apr 21;125(2):301-13

22. Pieters T, van Roy F2. Role of cell-cell adhesion complexes in embryonic stem cell biology. J Cell Sci. 2014 Jun 15;127(Pt 12):2603-13. doi: 10.1242/jcs.146720.
23. Altamirano J, Li Y, DeSantiago J, Piacentino V 3rd, Houser SR, Bers DM, The inotropic effect of cardioactive glycosides in ventricular myocytes requires Na+-Ca2+ exchanger function. J Physiol. 2006 Sep 15;575(Pt 3):845-54. Epub 2006 Jul 6.
24. Arispe N, Diaz JC, Simakova O, Pollard HB, Heart failure drug digitoxin induces calcium uptake into cells by forming transmembrane calcium channels. Proc Natl Acad Sci U S A. 2008 Feb 19;105(7):2610-5. doi: 10.1073/pnas.0712270105. Epub 2008 Feb 12.
25. Cavey M, Lecuit T. Molecular bases of cell-cell junctions stability and dynamics. Cold Spring Harb Perspect Biol. 2009 Nov;1(5):a002998. doi: 10.1101/cshperspect.a002998.
26. Gkountela S, Castro-Giner F, Szczerba BM, Vetter M, Landin J, Scherrer R, Krol I, Scheidmann MC, Beisel C, Stirnimann CU, Kurzeder C, Heinzelmann-Schwarz V, Rochlitz C, Weber WP, Aceto N. Circulating Tumor Cell Clustering Shapes DNA Methylation to Enable Metastasis Seeding.
27. Cell. 2019 Jan 10;176(1-2):98-112.e14. doi: 10.1016/j.cell.2018.11.046
28. McPherson A, Roth A, Laks E, Masud T, Bashashati A, Zhang AW, Ha G, Biele J, Yap D, Wan A, Prentice LM, Khattra J, Smith MA, Nielsen CB, Mullaly SC, Kalloger S, Karnezis A, Shumansky K, Siu C, Rosner J, Chan HL, Ho J, Melnyk N, Senz J, Yang W, Moore R, Mungall AJ, Marra MA, Bouchard-Côté A, Gilks CB, Huntsman DG1, McAlpine JN, Aparicio S, Shah SP. Divergent modes of clonal spread and intraperitoneal mixing in high-grade serous ovarian cancer. Nat Genet. 2016 Jul;48(7):758-67. doi: 10.1038/ng.3573. Epub 2016 May 16.
29. Reiter JG, Makohon-Moore AP, Gerold JM, Bozic I, Chatterjee K, Iacobuzio-Donahue CA, Vogelstein B, Nowak MA. Reconstructing metastatic seeding patterns of human cancers. Nat Commun. 2017 Jan 31;8:14114. doi: 10.1038/ncomms14114.
30. Wang J, Schwinger RH, Frank K, Müller-Ehmsen J, Martin-Vasallo P, Pressley TA, Xiang A, Erdmann E, McDonough AA. Regional expression of sodium pump subunits isoforms and Na+-Ca++ exchanger in the human heart. J Clin Invest. 1996 Oct 1;98(7):1650-8.
31. Biggar RJ, Wohlfahrt J, Oudin A, Hjuler T, Melbye M. Digoxin use and the risk of breast cancer in women. J Clin Oncol. 2011 Jun 1;29(16):2165-70. doi: 10.1200/JCO.2010.32.8146. Epub 2011 Mar 21
32. Xie SH, Jernberg T, Mattsson F, Lagergren J. Digitalis use and risk of gastrointestinal cancers: A nationwide population-based cohort study. Oncotarget. 2017 May 23;8(21):34727-34735. doi: 10.18632/oncotarget.16151.
33. 20 Osman MH, Farrag E, Selim M, Osman MS, Hasanine A, Selim A. Cardiac glycosides use and the risk and mortality of cancer; systematic review and meta-analysis of observational studies. PLoS One. 2017 Jun 7;12(6):e0178611. doi: 10.1371/journal.pone.0178611. eCollection 2017.
34. Menger L, Vacchelli E, Kepp O, Eggermont A, Tartour E, Zitvogel L, Kroemer G, Galluzzi L. Trial watch: Cardiac glycosides and cancer therapy. Oncoimmunology. 2013 Feb 1;2(2):e23082.
35. Goldin AG, Safa AR. Digitalis and cancer. Lancet. 1984 May 19;1(8386):1134
36. Stenkvist B. Is digitalis a therapy for breast carcinoma? Oncol Rep. 1999 May-Jun;6(3):493-6.
37. Guzman M, Gomez R, Romero SP, Aranda R, Andrey JL, Pedrosa MJ, Egido J, Gomez F.
38. Prognosis of heart failure treated with digoxin or with ivabradine: A cohort study in the community. Int J Clin Pract. 2018 Nov;72(11):e13217. doi: 10.1111/ijcp.13217. Epub 2018 Sep 24.
39. Lopes RD, Rordorf R, De Ferrari GM, Leonardi S, Thomas L, Wojdyla DM, Ridefelt P, Lawrence JH, De Caterina R, Vinereanu D, Hanna M, Flaker G, Al-Khatib SM, Hohnloser SH, Alexander JH, Granger CB, Wallentin L; ARISTOTLE Committees and Investigators. Digoxin and Mortality in Patients With Atrial Fibrillation. J Am Coll Cardiol. 2018 Mar 13;71(10):1063-1074. doi: 10.1016/j.jacc.2017.12.060.
40. Muzzarelli S, Stricker H, Pfister O, Foglia P, Moschovitis G, Mombelli G, Brunner-La Rocca H. Individual dosage of digoxin in patients with heart failure. QJM. 2011 Apr;104(4):309-17. doi: 10.1093/qjmed/hcq196. Epub 2010 Nov 8.

17. APPENDICES
