# Supplementary material for: Digoxin for reduction of circulating tumor cell cluster size in metastatic breast cancer: a proof-of-concept trial
Source: Nat Med. 2025 Jan 24;31(4):1120–4. doi: 10.1038/s41591-024-03486-6 (PMC12003195; doi:10.1038/s41591-024-03486-6)
Supplement: Supplementary file 2 — Reporting Summary [file 41591_2024_3486_MOESM2_ESM.pdf]

nature portfolio

Corresponding author(s): Nicola Aceto, PhD
Marcus Vetter, MD

Last updated by author(s): Dec 9, 2024

Reporting Summary

Nature Portfolio wishes to improve the reproducibility of the work that we publish. This form provides structure for consistency and transparency in reporting. For further information on Nature Portfolio policies, see our Editorial Policies and the Editorial Policy Checklist.

Statistics

For all statistical analyses, confirm that the following items are present in the figure legend, table legend, main text, or Methods section.

n/a	Confirmed	
☐	☒	The exact sample size (*n*) for each experimental group/condition, given as a discrete number and unit of measurement
☐	☒	A statement on whether measurements were taken from distinct samples or whether the same sample was measured repeatedly
☐	☒	The statistical test(s) used AND whether they are one- or two-sided<br>*Only common tests should be described solely by name; describe more complex techniques in the Methods section.*
☒	☐	A description of all covariates tested
☐	☒	A description of any assumptions or corrections, such as tests of normality and adjustment for multiple comparisons
☐	☒	A full description of the statistical parameters including central tendency (e.g. means) or other basic estimates (e.g. regression coefficient) AND variation (e.g. standard deviation) or associated estimates of uncertainty (e.g. confidence intervals)
☐	☒	For null hypothesis testing, the test statistic (e.g. *F*, *t*, *r*) with confidence intervals, effect sizes, degrees of freedom and *P* value noted<br>*Give P values as exact values whenever suitable.*
☒	☐	For Bayesian analysis, information on the choice of priors and Markov chain Monte Carlo settings
☒	☐	For hierarchical and complex designs, identification of the appropriate level for tests and full reporting of outcomes
☐	☒	Estimates of effect sizes (e.g. Cohen's *d*, Pearson's *r*), indicating how they were calculated

Our web collection on statistics for biologists contains articles on many of the points above.

Software and code

Policy information about availability of computer code

Data collection	No software was used for data collection.
Data analysis	Data analysis, statistical testing and visualization were conducted in Graphpad Prism (v.9.0.2) and R (version 4.2.2; R Foundation for Statistical Computing) and bioconductor (v.3.16). For RNA sequencing data quality control Trim Galore! (v0.6.6), FastQC (v0.11.9), FastQ Screen (v0.15.2) and MultiQC (v1.9) were used. For RNA sequencing alignment pipeline we used STAR (v.2.7.9a), Samtools (v1.10), featureCounts (v.2.0.3). For quality control, analysis, and visualization of processed RNA-seq data we used R/Bioconductor packages DESeq2 (v1.38.3), clusterProfiler (v4.6.0), ComplexHeatmap (v2.14.0). Code for RNA sequencing data analysis is available at https://github.com/TheAcetoLab/dicct-trial.

For manuscripts utilizing custom algorithms or software that are central to the research but not yet described in published literature, software must be made available to editors and reviewers. We strongly encourage code deposition in a community repository (e.g. GitHub). See the Nature Portfolio guidelines for submitting code & software for further information.

Data

Policy information about availability of data

All manuscripts must include a data availability statement. This statement should provide the following information, where applicable:

- Accession codes, unique identifiers, or web links for publicly available datasets
- A description of any restrictions on data availability
- For clinical datasets or third party data, please ensure that the statement adheres to our policy

Raw RNA sequencing data have been deposited and are publicly available in the Gene Expression Omnibus (GEO, NCBI; accession number GSE249233). Processed RNA sequencing data and other large data required for reproducibility are available from the Zenodo data repository (https://doi.org/10.5281/zenodo.10215050). Human reference genome (GRCh38) and human gene annotation (release 40) were downloaded from GENCODE (https://www.gencodegenes.org).

Research involving human participants, their data, or biological material

Policy information about studies with human participants or human data. See also policy information about sex, gender (identity/presentation), and sexual orientation and race, ethnicity and racism.

Reporting on sex and gender	Both male and female patients were considered eligible for the study. Female adult patients (n = 11) were included in the study. Separately, nine female patients (n = 9) with CTC clusters and matched clinical characteristics were non-randomly assigned to the untreated control group. Gender was not considered.
Reporting on race, ethnicity, or other socially relevant groupings	N/A
Population characteristics	Age ≥ 18 years, proven diagnosis of locoregionally recurrent or progressive metastatic breast cancer not amenable to curative treatment. Adequate organ and marrow function.
Recruitment	Eligible patients were recruited during routine clinical visits by a medical oncologist or a gynecologist. No specific bias in recruitment was identified. Patients meeting inclusion criteria were included after receiving detailed information on the study procedures and upon written informed consent. There was no participants compensation.
Ethics oversight	The study and related research projects were approved by the Swiss authorities Cantonal Ethics Committee Basel and Cantonal Ethics Committee Zurich in compliance with the Declaration of Helsinki (BASEC 2019-00673, BASEC 2021-01939, BASEC 2020-00014).

Note that full information on the approval of the study protocol must also be provided in the manuscript.

Field-specific reporting

Please select the one below that is the best fit for your research. If you are not sure, read the appropriate sections before making your selection.

☒ Life sciences ☐ Behavioural & social sciences ☐ Ecological, evolutionary & environmental sciences

For a reference copy of the document with all sections, see nature.com/documents/nr-reporting-summary-flat.pdf

Life sciences study design

All studies must disclose on these points even when the disclosure is negative.

Sample size	Clinical trial study design: it is expected that only 20-25 % of all patients in the study population have CTC clusters detectable in the peripheral circulation. Furthermore, it is expected that 20-25% of the patients will not reach the digoxin target level upon treatment. Therefore, the total number of patients included in the full analysis set (FAS) has been planned to be around 50-60. The sample size estimation was based on pilot data. For the primary outcome of the study, the comparison is conducted within each patient, therefore a paired test will be used. Based on this, the expected number of patients with a digoxin serum level within the target range after treatment is nine (80%), providing a power of 0.8 to estimate a mean treatment effect of digoxin of 1.1 (average CTC cluster size reduction, expressed in number of cells).<br>Animal study design: sample sizes were determined while adhering to 3R principles based on our previous experience (Diamantopoulou, Z. et al. The metastatic spread of breast cancer accelerates during sleep. Nature 607, 156–162 (2022); Szczerba, B. M. et al. Neutrophils escort circulating tumour cells to enable cell cycle progression. Nature 566, (2019)) and without predetermined calculations. Mice were randomized (without blinding) before each experiment.
Data exclusions	Two patients were excluded from the study: one of which due to the inability to reach the target digoxin serum level, and another due to a digoxin-unrelated adverse event.
Replication	The project is a prospective clinical trial and thus no replication was planned. Further studies planned to ultimately ensure reproducibility.
Randomization	The project is a single arm, proof-of-concept trial. The control cohort is non-randomized.

Blinding	The project is a single arm, proof-of-concept trial.

Reporting for specific materials, systems and methods

We require information from authors about some types of materials, experimental systems and methods used in many studies. Here, indicate whether each material, system or method listed is relevant to your study. If you are not sure if a list item applies to your research, read the appropriate section before selecting a response.

Materials & experimental systems

n/a	Involved in the study
☐	☒ Antibodies
☐	☒ Eukaryotic cell lines
☒	☐ Palaeontology and archaeology
☐	☒ Animals and other organisms
☐	☒ Clinical data
☒	☐ Dual use research of concern
☒	☐ Plants

Methods

n/a	Involved in the study
☒	☐ ChIP-seq
☒	☐ Flow cytometry
☒	☐ MRI-based neuroimaging

Antibodies

Antibodies used	anti-human EpCAM–AF488 (1:50; Cell Signaling Technology, CST5198), anti-human HER2–AF488 (1:50; BioLegend, 324410), anti-human EGFR–FITC (1:25; GeneTex, GTX11400), anti-human CD45-AF647 (1:25; BioLegend, 304018), anti-mouse CD45-AF647 (1:50; Biolegend, 103124).
Validation	According to the manufacture's website, each antibody was validated for its reactivity with the described human epitopes. We have previously validated same antibodies in human specimen (Diamantopoulou, Z. et al. The metastatic spread of breast cancer accelerates during sleep. Nature 607, 156–162 (2022); Szczerba, B. M. et al. Neutrophils escort circulating tumour cells to enable cell cycle progression. Nature 566, (2019).

Eukaryotic cell lines

Policy information about cell lines and Sex and Gender in Research

Cell line source(s)	4T1 murine breast cancer cells were purchased from ATCC (#CRL-2539).
Authentication	The cells were not authenticated.
Mycoplasma contamination	The cell line tested negative for mycoplasma contamination.
Commonly misidentified lines (See ICLAC register)	No commonly misidentified cell lines were used.

Animals and other research organisms

Policy information about studies involving animals; ARRIVE guidelines recommended for reporting animal research, and Sex and Gender in Research

Laboratory animals	8-12 -week-old NOD.Cg-Prkdcscid Il2rgtm1Wjl/SzJ (NSG) female mice were purchased from Charles River.
Wild animals	This study did not involve wild animals.
Reporting on sex	All animals included in this study were female in order to match the sex of the donors of the engrafted breast cancer cells.
Field-collected samples	This study did not involve samples collected from the field.
Ethics oversight	All mouse experiments were carried out according to institutional and cantonal guidelines (mouse protocol number 36338, approved by the cantonal veterinary office of Zurich).

Note that full information on the approval of the study protocol must also be provided in the manuscript.

Clinical data

Policy information about clinical studies

All manuscripts should comply with the ICMJE guidelines for publication of clinical research and a completed CONSORT checklist must be included with all submissions.

Clinical trial registration	NCT03928210
Study protocol	The full trial protocol is included in the submission.
Data collection	Recruitment and data collection for the digoxin-treated cohort and the non-randomized control cohort was performed between July 2020 and July 2024 at University Hospital Basel, Cancer Center Baselland, University Hospital Zurich.
Outcomes	Primary study outcome:<br>To assess the effect of digoxin on mean CTC cluster size.<br>Secondary study outcomes:<br>To assess the effect of digoxin on mean CTC cluster number.<br>To assess the effect of digoxin on mean time to dissolution of CTC clusters.

Plants

Seed stocks	N/A
Novel plant genotypes	N/A
Authentication	N/A
